# Outer Membrane Vesicles of *Acinetobacter baumannii* DS002 Are Selectively Enriched with TonB-Dependent Transporters and Play a Key Role in Iron Acquisition

**DOI:** 10.1128/spectrum.00293-22

**Published:** 2022-03-10

**Authors:** Ganeshwari Dhurve, Ashok Kumar Madikonda, Medicharla Venkata Jagannadham, Dayananda Siddavattam

**Affiliations:** a Dept. of Animal Biology, School of Life Sciences, University of Hyderabad, Hyderabad, India; b Metabolomics Facility, School of Life Sciences, University of Hyderabad, Hyderabad, India; University of Manitoba

**Keywords:** proteomics, OMVs, LC-ESI-MS/MS, TonRs, iron uptake, siderophores

## Abstract

Outer membrane vesicles (OMVs) of Acinetobacter baumannii DS002 carry proteins which perform selective biological functions. The proteins involved in cell wall/membrane biogenesis and inorganic ion transport and metabolism occupied a significant portion of the 302 proteins associated with OMVs. Interestingly, the TonB-dependent transporters (TonRs), linked to the active transport of nutrients across the energy-deprived outer membrane, are predominant among proteins involved in inorganic ion transport and metabolism. The OMVs of DS002 contain TonRs capable of transporting iron complexed to catecholate, hydroximate, and mixed types of siderophores. Consistent with this observation, the OMVs were firmly bound to ferric-enterobactin (^55^Fe-Ent) and successfully transported iron into A. baumannii DS002 cells grown under iron-limiting conditions. In addition to the TonRs, OMVs also carry proteins known to promote pathogenesis, immune evasion, and biofilm formation. Our findings provide conclusive evidence for the role of OMVs in the transport of nutrients such as iron and show the presence of proteins with proven roles in pathogenicity and immune response.

**IMPORTANCE** TonB-dependent transporters (TonRs) play a crucial role in transporting nutrients such as iron, nickel, copper, and complex carbohydrates across the energy-deprived outer membrane. Due to their unique structural features, TonRs capture nutrients in an energy-independent manner and transport them across the outer membrane by harvesting energy derived from the inner membrane-localized Ton-complex. In this study, we report the presence of TonRs capable of transporting various nutrients in OMVs and demonstrate their role in capturing and transporting ferric iron complexed with enterobactin into A. baumannii DS002 cells. The OMV-associated TonRs appear to play a critical role in the survival of A. baumannii, listed as a priority pathogen, under nutrient-deprived conditions.

## INTRODUCTION

Outer membrane vesicles (OMVs) of 20 to 300 nm in diameter, released by Gram-negative bacteria, play a crucial role in the survival of bacterial cells under adverse environmental conditions (1). They play critical roles in nutrient acquisition, pathogenesis, quorum-sensing signaling, and horizontal gene transfer (2). Almost all Gram-negative bacteria, including those infecting host tissue and serum, produce OMVs (3). OMVs contribute to the survival of bacterial cells by performing several critical cellular activities. They remove misfolded proteins, perform transport functions, and carry enzymes that generate carbon sources from complex macromolecules (3). The OMVs also contain signaling molecules that influence cellular communication and host immune response (1). Despite significant clues on the physiological relevance of OMVs, no conclusive evidence is available regarding their biogenesis (4). It is widely believed that the tiny blebs formed due to the presence of curve-induced proteins in the outer membrane slowly dissociate to form these spherical nanostructures (5). These blebs are also developed in the outer membrane due to the accumulation of certain proteins in the periplasmic space, and during delinking of the outer membrane from the damaged peptidoglycan (6). While OMVs come out of the outer membrane, these blebs carry certain periplasmic and outer membrane proteins that play crucial roles in pathogenesis, quorum signaling, and nutrient acquisition. OMVs also carry DNA and play a key role in horizontal gene transfer (7).

A. baumannii cells survive in a variety of habitats. They live in soil, water, and on the surfaces of human and animal bodies as free-living organisms. Certain strains of A. baumannii quickly adapt to a pathogenic lifestyle and cause nosocomial infections (8). A. baumannii strains survive longer on dry surfaces, such as hospital beds and surgical instruments. It is one of the leading opportunistic pathogens among Gram-negative bacteria which infect immunocompromised patients admitted to critical care units (9). A. baumannii strains were susceptible to commonly used antibiotics like gentamicin, minocycline, nalidixic acid, ampicillin, and carbenicillin (10). During the 1970s, these antibiotics were used either singly or in combination to treat A. baumannii infections. However, by late 1990, carbapenems became the only useful drugs to control Acinetobacter infections (11). A. baumannii is now included in the list of priority pathogens by the World Health Organization (WHO) due to the limited therapeutic options available to control its infections (11).

A. baumannii DS002 is a soil isolate and survives in pesticide-polluted agriculture soils by thriving on toxic phenolic waste (8). Genome information is available for strain DS002, and it contains a chromosome (3,430,799 bp) and six indigenous plasmids (8, 12). We have recently compared the genome of DS002 with genome sequences of A. baumannii strains isolated from different habitats (8). Interestingly, we have noticed the presence of genes coding several TonB-dependent transporters (TonRs) involved in the transport of iron complexed with various siderophores. However, the genes coding the corresponding siderophores are absent in the genome of A. baumannii DS002. Since OMVs have a proven role in transporting nutrients, we examined whether OMVs use these TonRs for iron acquisition. Our studies demonstrated the selective enrichment of TonRs in OMVs of A. baumannii DS002 and their role in iron acquisition.

## RESULTS

The TonB-dependent transporters (TonRs) actively transport several nutrients, including iron, across the energy-deprived outer membrane of Gram-negative bacteria (13). They use energy generated by the inner membrane-associated Ton-complex while transporting siderophores complexed to iron across the outer membrane. The genome of A. baumannii DS002 codes for several TonRs, which transport all three major types of siderophores. Intriguingly, the genetic repertoires required for the synthesis of corresponding siderophores are absent in the genome of DS002 (8). In the absence of siderophores, DS002 cells must acquire iron using siderophores synthesized and secreted by cohabiting bacteria. Outer membrane vesicles (OMVs) play a critical role in meeting the nutritional requirements of the host. They carry various nutrients, including iron, due to the presence of membrane transporters and iron-binding proteins. This study is primarily designed to identify TonRs in the OMVs of DS002 and unravel their role in transport of iron.

**FIG 1:**
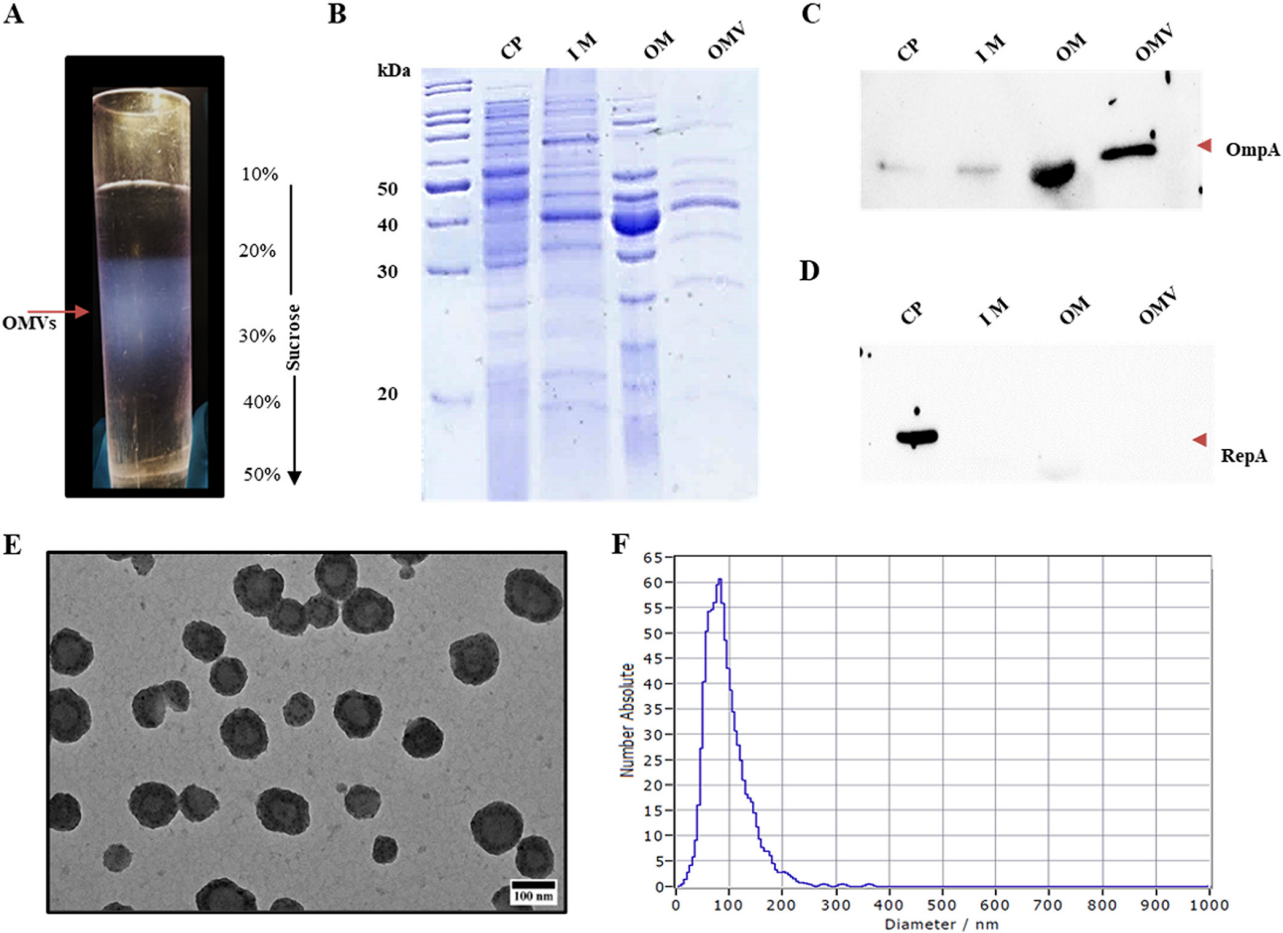
Purification of outer membrane vesicles (OMVs) from Acinetobacter baumannii DS002. (A) OMV band on sucrose density gradient (10 to 50%). (B) SDS-PAGE (12%) showing the profiles of cytoplasmic (CP), inner membrane (IM), outer membrane (OM), and OMV proteins. Corresponding Western blots, developed by probing with either OmpA or RepA antibodies, are shown in panels C and D, respectively. Panel E shows transmission electron microscopy (TEM) images of pure OMVs, showing their size distribution. Size distribution of OMVs as measured by particle matrix analyser (ZetaView) is shown in panel F.

Initially, we purified the OMVs using sucrose density gradient ultracentrifugation (Fig. 1A) and tested their purity by detecting marker proteins specific to OMVs. Outer membrane porin, OmpA, plays critical roles in biogenesis and maintaining the integrity of OMVs (14). Since it is exclusively present in the outer membrane, we have used it as an OMV-specific marker. RepA, involved in the initiation of DNA replication, is used as a cytoplasmic protein marker (15). SecYEG, the inner membrane-associated protein-conducting channel, served as an inner membrane-specific marker (16). At first, we analyzed OMV, cytoplasmic, and inner and outer membrane proteins with SDS-PAGE, and later probed them with the antibodies of maker proteins to assess the purity of the OMVs (Fig. 1C and D). The SDS-PAGE profiles of OMVs and outer membrane proteins shared significant similarities. Most of the protein bands seen in OMVs were also found in the outer membrane (Fig. 1B, lanes 4 and 5). When these proteins were probed independently with antibodies against OmpA and RepA, the RepA-specific signal was exclusively seen in the lane loaded with cytoplasmic proteins (Fig. 1D, lane 1). The RepA-specific signals were not seen in lanes loaded with proteins extracted from OMVs and the outer membrane, suggesting that these fractions were not contaminated with cytoplasmic proteins (Fig. 1D, lanes 2, 3, and 4). Inferring that the isolated OMVs are free from cytoplasmic fraction, the OmpA-specific signals were only seen in lanes loaded with OM and OMV proteins, not in the lane loaded with cytoplasmic proteins (Fig. 1C, lanes 1, 2, 3, and 4). The OMVs isolated from the A. baumannii DS002 were also free from the inner membrane fraction. The SecYEG protein complex was not detected in the list of total OMV proteins identified by liquid chromatography electrospray ionization-tandem mass spectrometry (LC-ESI-MS/MS) using a Q Exactive mass spectrometer (Table S1 in the supplemental material).

After ascertaining the purity of isolated OMVs of A. baumannii DS002, we obtained electron micrographs for the negatively stained, purified OMVs and measured the mean and median diameters, range, and skew of the OMVs isolated from A. baumannii. The isolated OMVs have a mean diameter of 120 nm and range from 20 nm to 300 nm (Fig. 1E and F). The size distribution profiles of OMVs isolated from three biological replicates were independently determined as described in the Methods section. As seen in Fig. 1E, OMVs range from 74 nm to 160 nm. The isolated OMVs from A. baumannii DS002 are significantly similar in size to those isolated from A. baumannii ATCC 19696 (17).

### Analysis of OMV proteome from *A. baumannii*.

**FIG 2:**
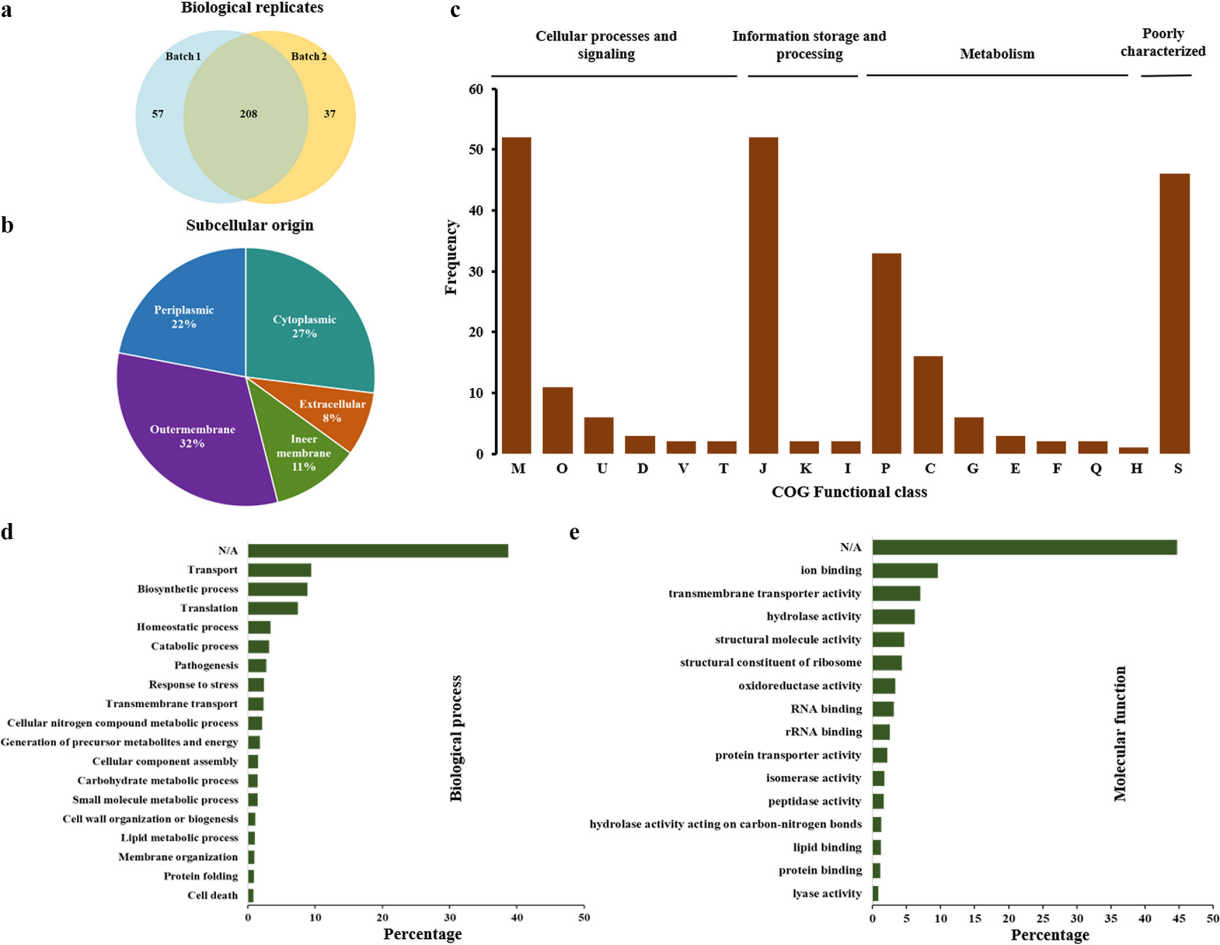
Analysis of OMV proteins. (a) Venn diagram showing the total 302 OMV proteins identified from two biological replicates. (b) Pie chart indicating subcellular origin of OMV proteins as predicted by PSORTb version 3. and CELLO2GO. (c) Cluster of orthologous group (COG) categorization of OMV proteins, where letters indicate proteins performing various functions: M, cell wall/membrane/envelope biogenesis; O, posttranslational modification, protein turnover, and chaperones; U, intracellular trafficking, secretion, and vesicular transport; D, cell cycle control, cell division, and chromosome partitioning; V, defense mechanisms; T, signal transduction mechanisms; J, translation, ribosomal structure and biogenesis; K, transcription; I, lipid transport and metabolism; P, inorganic ion transport and metabolism; C, energy production and conversion; G, carbohydrate transport and metabolism; E, amino acid transport and metabolism; F, nucleotide transport and metabolism; Q, secondary metabolites biosynthesis, transport, and catabolism; H, coenzyme transport and metabolism; and S, function unknown. Gene ontology based on functional classifications, biological processes, and molecular functions of OMV proteins is shown in panels d and e, respectively.

While gaining more insights into the identity of proteins associated with OMVs, we initially performed matrix-assisted laser desorption ionization–tandem time of flight (MALDI-TOF/TOF) analysis. The lane containing OMV proteins was divided into five zones, and the proteins present in each zone were identified by determining the peptide mass fingerprint and tandem mass spectrometry (MS-MS) analysis, as detailed in the Methods section (Fig. 1B, lane 5). The prominent protein band in the lane was identified as OmpA by performing both MALDI and ESI-MS/MS studies. The rest of the proteome of the OMVs was identified using LC-ESI-MS/MS analysis. OMVs isolated from two independent culture batches were used to minimize detection errors and improve the identity of the number of proteins in OMVs. In total, 302 proteins were detected in OMVs isolated from A. baumannii DS002 (Fig. 2a). Of these 302 proteins, the identities of 265 and 245 proteins were established from OMVs isolated from the first and second batches, respectively (Fig. 2a). Interestingly, among these 302 proteins, 32% were from the outer membrane and 22% were periplasmic proteins. Detection of a large number of outer membrane and periplasmic proteins in OMVs reconfirms the purity of the isolated OMVs (Fig. 2b). After establishing the identity of the OMV proteins, we clustered them into orthologous groups (COG). The names of these 302 proteins and their putative functions are given in Table S1. Out of these 302 proteins, only 254 proteins could be assigned a function. The COG clustering included 76 OMV proteins associated with cellular process and signaling, 63 with metabolism, and 56 with information storage and processing. Surprisingly, 46 proteins were identified within the group of poorly characterized proteins (Fig. 2c). The COG categorization showed that the majority of OMV proteins are related to inorganic ion transport and storage, cell wall/membrane biogenesis, and pathogenesis (Fig. 2c). Further studies conducted on gene ontology revealed that most OMV proteins are involved in biological processes, such as transport, translation, and pathogenesis (Fig. 2d). Ion binding, transmembrane transport, and RNA binding appear to be the main molecular functions of OMV proteins (Fig. 2e).

### TonB-dependent transporters were enriched in OMVs.

A majority of OMV proteins performing transport functions are TonB-dependent transporters (TonRs). TonRs play a key role in the active transport of nutrients across energy-deprived outer membranes. TonRs are different from outer membrane porins. Substrates pass through porins by diffusion; whereas in TonRs, nutrients exceeding 600 Da in size cross the outer membrane by utilizing the energy generated by the inner membrane-localized Ton-complex (18). The Ton-complex contains ExbB/ExbD and TonB, where the proton motive force (PMF) components, ExbB/ExbD, generate energy and TonB harvests energy and transduces it to outer membrane-localized TonRs (19, 20). TonRs have unique structural features that are very distinct from porins. The C-terminal region of TonR contains 22 antiparallel β-strands which form a membrane-spanning barrel domain, and it is significantly bigger than the barrel domain of porins (21). In addition, the TonRs contain an N-terminal plug domain which obstructs the passage of solutes. Substrates to be transported are specifically recognized by the plug domain or by the external loops of the barrel, independent of the involvement of the energy transducer TonB (22). However, the translocation of substrate into periplasmic space requires a functional Ton-complex. The five-amino acids-long TonB box motif (ETVIV) found at the plug domain interacts with the C-terminal domain of the energy transducer TonB to obtain the energy required for transporting a substrate bound to plug domain (23, 24).

**FIG 3:**
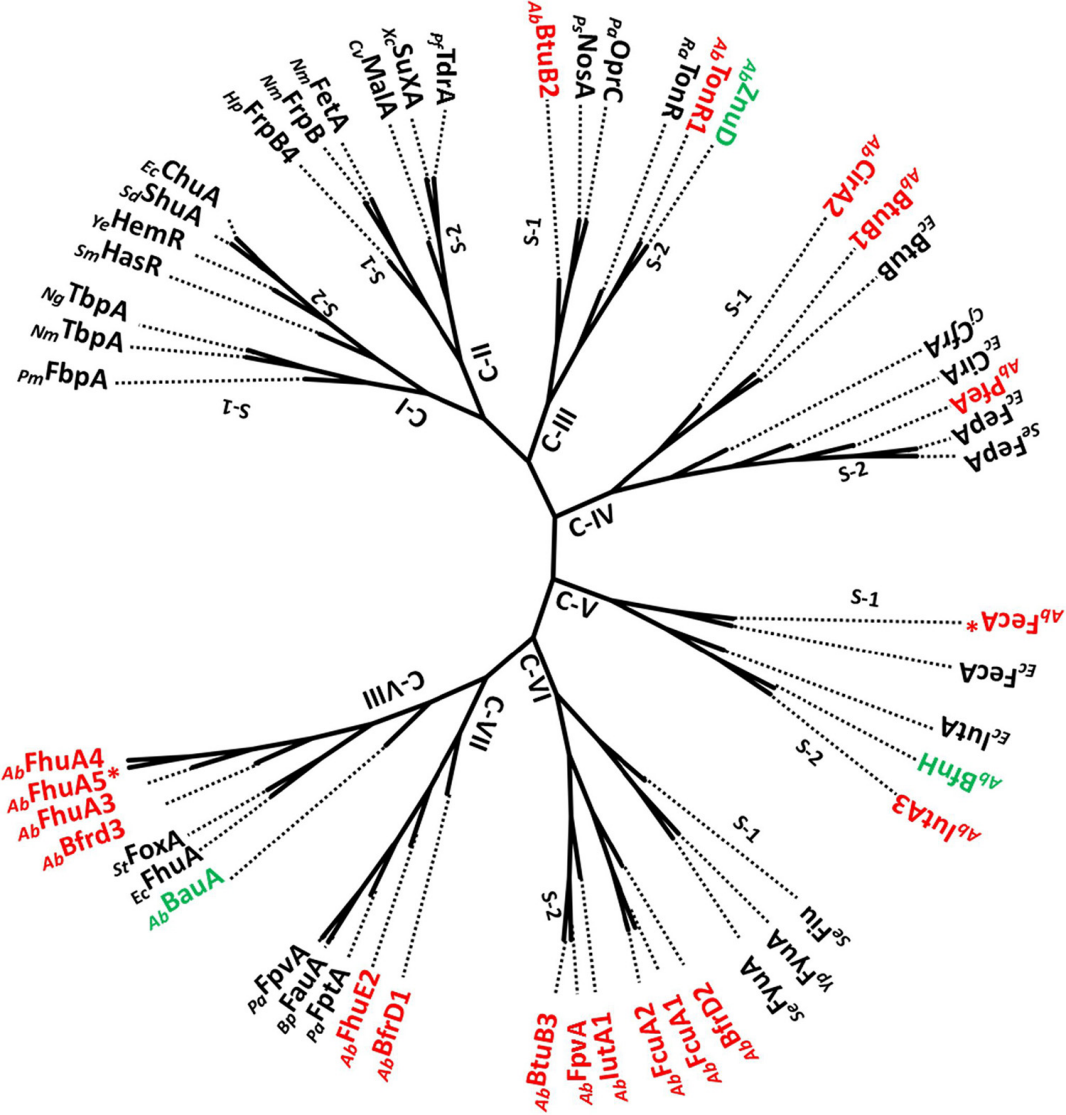
Unrooted phylogram constructed by including functionally characterized TonB-dependent transporters (TonRs) along with TonRs of OMVs of A. baumannii DS002. TonRs in black font are functionally characterized. TonRs in green font are functionally characterized TonRs from A. baumannii strains. OMV-associated TonRs are shown in red font. Plasmid-coded TonRs are indicated with an asterisk (*). TonRs of Pasteurella multocida (*_Pm_*FbpA), Neisseria meningitidis (*_Nm_*TbpA, *_Nm_*FrpB, *_Nm_*FetA), Neisseria gonorrhoeae (*_Ng_*TbpA), Serratia marcescens (*_Sm_*HasR), Yersinia enterocolitica (*_Ye_*HemR), Shigella dysenteriae (*_Sd_*ShuA), Escherichia coli (*_Ec_*ChuA, *_Ec_*BtuB, *_Ec_*CirA, *_Ec_*FepA, *_Ec_*FecA, *_Ec_*IutA, *_Ec_*FhuA,), Helicobacter pylori (*_Hp_*FrpB4), Caulobacter vibrioides, (*_Cv_*MalA), Xanthomonas campestris (*_Xc_*SuxA), Pseudomonas fluorescens (*_Pf_*TdrA), Pseudomonas stutzeri (*_Ps_*NosA), Pseudomonas aeruginosa (*_Pa_*OprC, *_Pa_*FptA, *_Pa_*FpvA), Riemerella anatipestifer (*_Ra_*TonR), Campylobacter jejuni (*_Cj_*CfrA), Salmonella*_enterica* (*_Se_*Fiu, *_Se_*FyuA), Yersinia pestis (*_Yp_*FyuA), Bordetella pertussis (*_Bp_*FauA), and Salmonella Typhimurium (*_St_*FoxA) are included in constructing the phylogram.

The EggNog mapper identified 24 OMV proteins as TonRs (Table S2). These TonR sequences were then reexamined to verify whether the OMV-associated TonRs had sequence motifs typically seen in well-characterized TonRs. Out of 24 TonR sequences, only 19 were full-length TonRs, and the rest of the 5 had either C-terminal or N-terminal truncations. We have taken only 19 full-length TonRs of OMVs for further analysis. Since TonRs are known to transport many nutrients in addition to iron, we have followed two independent approaches to ascertain the putative functions of OMV-associated TonRs. In the first approach, we have constructed a phylogenetic tree for OMV-associated TonRs by including functionally characterized TonR sequences (25). Based on the cladding pattern, we have assigned putative functions to the TonRs of OMVs (Fig. 3). These results were further verified by assessing the coregulation of genes coding these TonRs.

The generated phylogram contained eight clades, numbered from C-I to C-VIII (Fig. 3). Interestingly, clades C-I and C-II represent TonRs of pathogenic bacteria, transporting ferritin, heme, and carbohydrates such as maltodextrin and sucrose. Interestingly, none of the OMV TonRs of DS002 are found in these two clades. Most of the TonRs of OMVs of DS002 are identified in clades C-VI, C-VII, and C-VIII. The clade C-VI contains six OMV TonRs, and they have clustered with TonRs transporting both catecholate (Fiu) and phenolate (FyuA) type siderophores. Incidentally, all OMV TonRs of DS002 have branched out to form a separate subclade, suggesting that they are unique from TonRs transporting phenolate and catecholate type siderophores (23). Clades V-II and V-III represent six TonRs of DS002, two of which formed clade C-VII by clustering with TonRs transporting pyochelin (FptA), alcaligin (FauA), and pyoverdine (FpvA), and the remaining four DS002 TonRs formed clade C-VIII by aligning well with FoxA and FhuA, which transport iron complexed with ferrioxamine (FoxA) and ferrichrome (FhuA). The cladding pattern suggests that the six TonRs present in these two clades might also transport iron complexed with five deferent types of siderophores. In addition to iron transporters, clade C-III, with its two subclades, contained both copper- and zinc-transporting TonRs. There are two DS002 TonRs in these two subclades: BtuB2, aligned with copper transporters NosA and OprC (26, 27); and TonR1, with zinc transporter ZnuD (28). Likewise, clade C-IV also contains two subclades, and in one of them, two DS002 TonRs, BtuB1 and CirA, have clustered with cobalamin-transporting BtuB (29). Subclade 2 of clade C-IV contains one DS002 TonR, which seems to align well with the ferric enterobactin (Fe-Ent) transporter, FepA. Clade C-V, with five TonRs is divided into two sub clades. In subclade S1, plasmid-encoded TonR (*_Ab_*FecA*) is clustered with FecA of E. coli. Similarly, in subclade S2, OMV TonR, annotated as *_Ab_*IutA3, is clustered with BfnH of A. baumannii ATCC 19606. BfnH transports iron bound to acinetobactin (30, 31). _Ab_IutA3 seems to have a role in the transport of acinetobactin complexed with iron.

**FIG 4:**
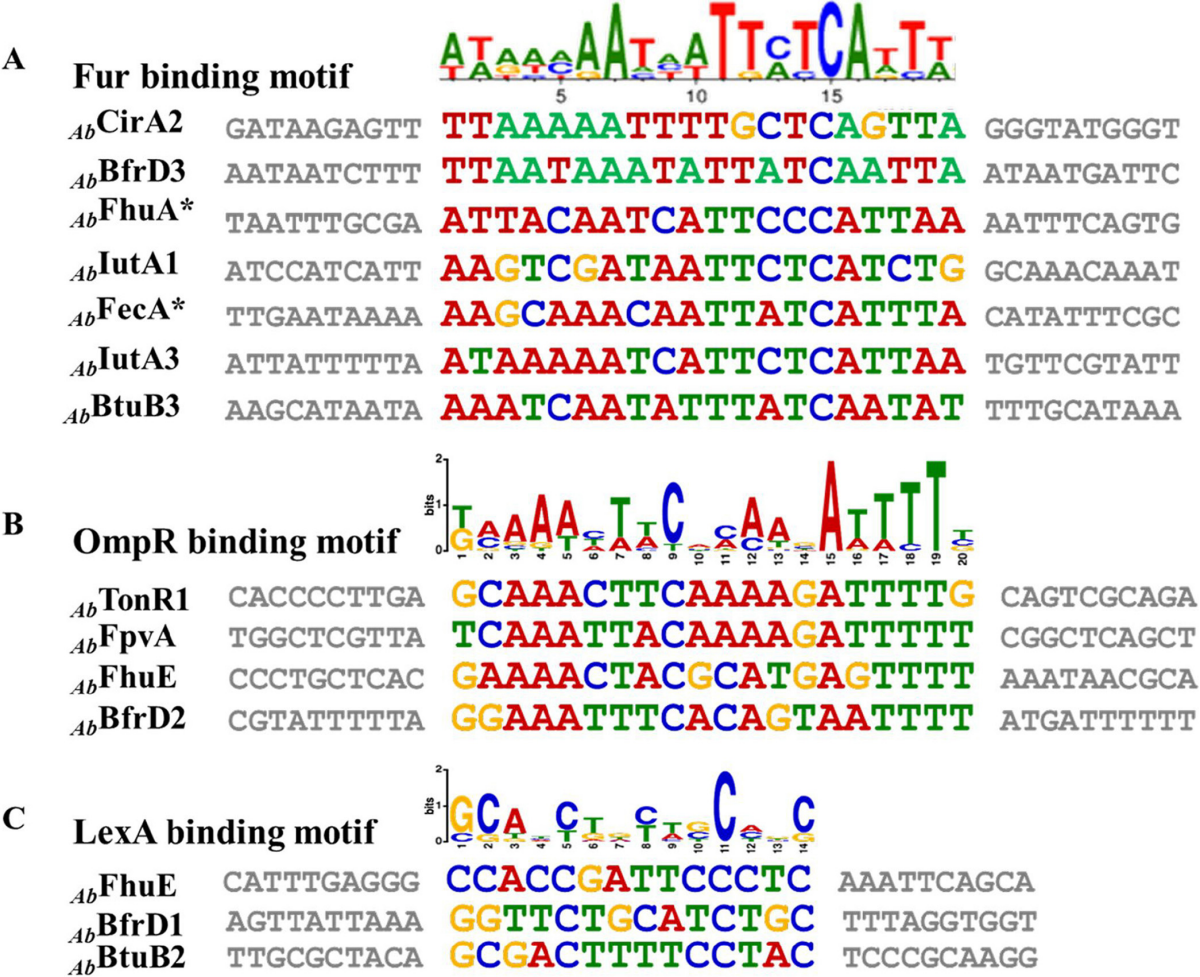
Predicted transcription factor-binding motifs overlapping TonR-coding genes. (A) *fur*-box motif predicted overlapping the promoters of genes encoding *_Ab_*CirA2, *_Ab_*BfrD3, *_Ab_*FhuA*, *_Ab_*IutA1, *_Ab_*FecA*, *_Ab_*IutA3, and *_Ab_*BtuB3. The OmpR-binding sites identified overlapping *_Ab_*FpvA, *_Ab_*FhuE, and *_Ab_*BfrD2, and LexA-binding sites found at the promoters of *_Ab_*FhuE-, *_Ab_*BfrD1,- and *_Ab_*BtuB2-coding genes, are shown in panels B and C, respectively.

In the generated phylogram, some of the TonRs of OMVs have clustered with functionally characterized TonB-dependent transporters. These TonRs transport iron complexed with many different types of siderophores. Such alignment of OMV proteins with functionally characterized TonRs suggests their involvement in iron transport. Proteins involved in iron acquisition are coded by genes regulated by ferric uptake regulatory protein (Fur). Fur is a dual-transcription regulator and regulates genes in response to the intracellular iron concentration (32). Since Fur binds to the conserved *fur*-box motif, we have examined the promoters of TonR-coding genes to identify *fur*-box motif (33). Our analysis revealed the presence of a well-conserved *fur*-box overlapping the promoters of seven TonR-coding genes (Fig. 4). In addition to the *fur*-box motif, we have also noticed conserved sequence motifs overlapping the promoters of certain TonR-coding sequences which serve as targets for the transcription factors LexA and OmpR. These two transcription factors respond to oxidative stress and osmolarity, respectively (34, 35). Iron deficiency is known to induce oxidative stress, and LexA and OmpR counter this stress by modulating the expression of several genes (35). However, the existence of TonRs under the control of LexA and OmpR is rather unusual. The small RNAs OmrA/OmrB, which downregulate TonRs such as CirA, FecA, and FepA, are regulated by OmpR (36). The phosphorylated form of OmpR, produced in response to osmolarity of the external medium, positively regulates *omrA*/*omrB*. Nonetheless, the link between osmotic stress and the expression of CirA, FecA, and FepA is unclear. Further studies are required to explain the relationship between TonR expression and extracellular osmolarity.

### OMVs transport iron complexed with enterobactin.

**FIG 5:**
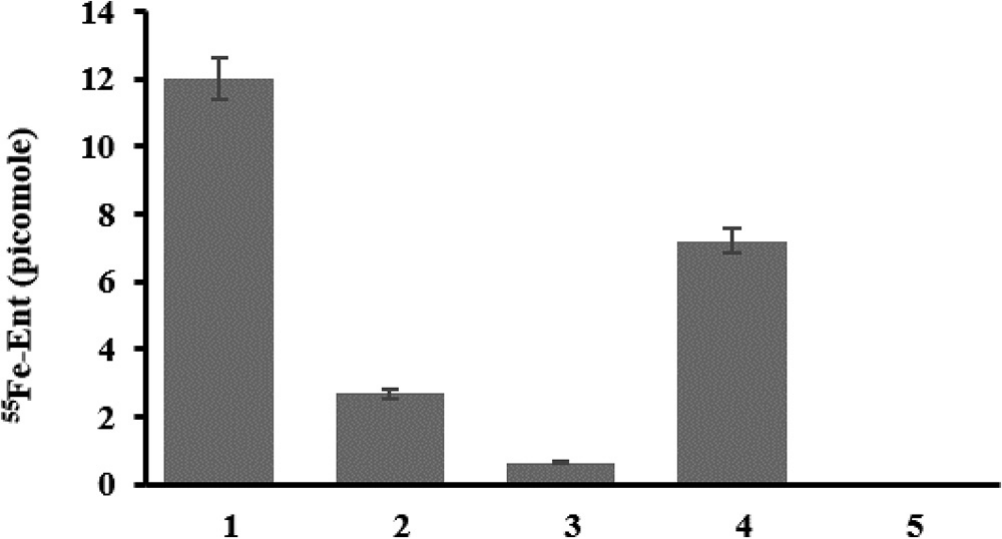
OMV-assisted iron uptake in A. baumannii DS002. Lane 1 indicates the amount of ^55^Fe-Ent bound to OMVs. After incubating with OMVs associated with ^55^Fe-Ent, the cells were collected and washed with lithium chloride to remove surface-associated ^55^Fe-Ent. The unbound and cell surface-associated ^55^Fe-Ent are shown in lanes 2 and 3, respectively. Lane 4 shows ^55^Fe present inside the cells after washing with lithium chloride. The control (unlabeled OMVs incubated with cells) is shown in lane 5.

The presence of large number of iron-transporting TonRs in nanostructures like OMVs is expected to increase surface area, enabling them to efficiently capture siderophores. We have tested this proposition by using radiolabeled ferric iron (^55^Fe) complexed with enterobactin (Ent). Initially, we prepared pure ferric-enterobactin (Fe-Ent) by incubating commercially procured enterobactin with ^55^Fe (American Radiolabeled Chemicals, ARX-0109). The purified ^55^Fe-Ent was then incubated with OMVs, as described in the Methods section, and repurified following density gradient centrifugation. Since the binding of ^55^Fe-Ent requires no energy, Fe-Ent bound strongly with the OMVs. The bound ^55^Fe-Ent did not dissociate from OMVs, even after the OMVs were repurified through density gradient centrifugation. About 72 pmol of ^55^Fe was bound to 200 μg of OMVs (Fig. 5). The OMVs complexed with ^55^Fe-Ent were then used to test whether they can transport iron into DS002 cells grown under iron-limiting conditions. We incubated 34 μg of OMVs (12 pmol of ^55^Fe) with 8 × 10^8^ cells for 12 h and used these cells to examine whether radiolabeled iron was found inside the cells. The surface-accumulated iron was removed by washing the cells with lithium chloride, and the actual amount of ^55^Fe translocated into the cytoplasm was measured as described in the Methods section. About 7.2 pmol iron was transported into DS002 cells by the 34 μg OMVs. These results clearly demonstrate the ability of OMVs to transport iron into DS002 cells by utilizing Fe-Ent. TonRs of OMVs have aligned with TonRs transporting iron bound to all three major classes of siderophores. OMVs also have TonRs that aligned well with zinc (*_Ab_*TonR1) and copper (*_Ab_*ButB2) transporters (Fig. 3). If these are available in the vicinity, the OMVs might also capture and transport them into A. baumannii DS002 cells along with iron and other nutrients.

### EF-TU is among the OMV-associated proteins.

OMVs of DS002 also carry proteins typically seen in OMVs of pathogenic bacteria. Proteins such as elongation factor Tu (EF-Tu), curli protein transporter, CsgG, and peptidyl prolyl *cis-trans* isomerase are seen among the OMV proteome (Table S1). Identification of EF-Tu with outer membrane and OMVs is not uncommon. A number of studies have highlighted its existence in OMVs and its moonlighting activities (37, 38). EF-Tu is known to interact with immune system regulators such as Factor H, substance P, and plasminogen, and thus increases virulence by helping immune system evasion (39). Recent studies have also demonstrated a decreased bacterial load in subjects with antibodies against EF-Tu (39). EF-Tu has also been shown to interact with fibronectins and glycosaminoglycans to facilitate adhesion of bacteria to human cells (37). Peptidyl-prolyl isomerases (PPIs) play a key role in protein folding by catalyzing the *cis*-*trans* isomerisation of peptide bonds N-terminal to proline residues. The isomerization process contributes to the mechanical properties of materials required to generate various extracellular matrices (40). Extracellular matrices are also generated during biofilm formation. Therefore, the association of EF-Tu and PPI with OMVs, which have a proven role in biofilm formation, is not surprising.

## DISCUSSION

In Gram-negative bacteria, the outer membrane hinders the uptake of nutrients which exceed the pore size of outer membrane porins (41). Such nutrients, and nutrients which are scarcely available in the environment, require active transport. Active transport across energy deprived outer membrane is dependent on the inner membrane-associated Ton-complex comprised of the proton motive force components ExbB/ExbD and TonB (19). The TonB harvests energy from PMF components and transduces it to the outer membrane-located TonR. This TonR uses that energy to translocate bound substrates across the outer membrane (19). The outer membrane active transport system, otherwise known as the TonB-dependent-transport (TBDT) system, was discovered during investigation of the details of phage T1 infection (42). Its roles have been subsequently shown in the transport of vitamin B_12_ and iron complexed with siderophores (43). Several studies have linked the TBDT system with iron acquisition and shown that the expression of TonR-coding genes is under the transcriptional control of ferric iron uptake regulator protein (32). Therefore, the TBDT system became synonymous with iron acquisition. However, recent studies have demolished this myth and shown that TonB-energized transport is also required for the transport of nickel, copper, zinc, and carbohydrates such as maltodextrins and sucrose (43). In fact, the expression of a nickel-specific TonR, FrpB4, of the human pathogen Helicobacter pylori, is strongly regulated by nickel ions (44, 45). Likewise, the expression of MalA is upregulated only when maltodextrins were used as sole source of carbon (46). Genomes of Gram-negative bacteria contain several TonR-coding sequences, and their number increases with the complexity of their habitat (43). The TonRs are proposed to have roles in the transport of complex nutrients and to facilitate organismal survival in complex environments.

Gram-negative bacteria, without exception, produce OMVs. The cargo carried by OMVs contains proteins and secondary metabolites that perform various cellular activities (3). OMV-associated proteins influence pathogenesis, immune response, signaling activities, and transport functions (47). Interestingly, the OMVs isolated from A. baumannii DS002 contain 19 different TonRs. Of these, FecA and FhuA5 are coded by the large indigenous plasmid pTS134338. The genomic island involved in iron acquisition contains two TonRs, FcuA1 and FcuA2, and the rest of the TonR-coding sequences have been identified on the chromosome (Fig. 3). Among these TonRs, only seven are under the transcriptional control of Fur protein. These Fur-regulated TonRs showed structural similarities to the TonRs involved in the transport of iron complexed with siderophores such as enterobactin, aerobactin, alcaligin, and hydroxy carboxylates (Fig. 3). Due to the presence of these TonRs, the OMVs are expected to capture different types of siderophores synthesized and secreted by cohabiting bacteria and use them to transport ferric iron into A. baumannii DS002. Thus, the OMV-mediated transport mechanism contributes to the survival of A. baumannii DS002, especially in a nutrient-limiting polymicrobial environment.

The true physiological significance of the TonB-dependent transport (TBDT) system is slowly unfolding (23). Besides iron, the TBDT system facilitates the transport of several nutrients and carbon sources (13). The presence of multiple TonRs in nanostructures, such as OMVs, serves to capture nutrients and carbon resources which are scarcely available in the environment. Since ligand binding to the plug domain of the receptor is energy independent, the TonRs load nutrients/carbon sources onto OMVs (4, 47, 48). These nutrient-loaded OMVs, when fused to the outer membrane of A. baumannii, gain access to the inner membrane-located Ton-complex to gain the energy required to translocate the nutrients into the periplasmic space.

Several studies have highlighted the role of OMVs in intra- and interspecies delivery (49–51). Once released from their mother cells, OMVs travel to distant places and deliver associated macromolecules to species that share no obvious taxonomic relationship (52). If these findings are viewed together with the structural diversity of OMV-associated TonRs, the role of OMVs in meeting the nutrient requirements of the microbial community is evident. These nanostructures, with increased surface area and TonR diversity, promote the survival of the microbial community by capturing and delivering scarcely available nutrients, including iron. The COG categorization included nearly 50% of the OMV-associated proteins which have unknown functions. Unless their functions are known, it is hard to realize the complete role of OMVs in the physiology and adaptive potential of A. baumannii DS002.

## MATERIALS AND METHODS

### Bacterial strains and plasmids.

Bacterial strains, plasmids, and primers used in this study are shown in Table S3. Acinetobacter baumannii DS002 was grown at 30°C in Luria Bertani (LB) medium. Gene manipulation, cloning, expression, and Western blotting were performed following standard procedures (53, 54). Biochemicals and restriction enzymes used in this study were purchased from Thermo Fisher Scientific India (Powai, India) and used following the manufacturers’ protocols.

### Isolation of outer membrane vesicles.

OMVs were isolated from A. baumannii DS002 culture supernatants following methods described elsewhere, with minor modifications (55). Briefly, A. baumannii DS002 cultures (4 L) were grown at 30°C until the culture optical density at 600 nm (OD_600_) reached 1.8, and cells were harvested by centrifuging the culture at 10,000 × *g* for 30 min. The cell pellet was flash-frozen and stored at −80°C until it was used for preparing subcellular fractions following standard procedures (56). The culture supernatant was taken into a sterile container and appropriate amounts of 500 mM EDTA (pH 8.0) stock was added until the EDTA concentration reached 1 mM. The supernatant was then passed through a 0.45-μm vacuum filter (Millipore) to remove residual cells and cell debris. The cell-free culture supernatant was concentrated to 600 mL by passing it through a 100-kDa hollow fiber membrane (GE Healthcare). The concentrated culture supernatant was again passed through a 0.45-μm filter before centrifuging (Rotor Type Ti70) at 150,000 × *g* for 2 h to pellet the OMVs. The OMVs were resuspended in 1 mL phosphate-buffered saline (PBS) buffer (pH 7.3), and carefully layered on a centrifuge tube containing a sucrose gradient ranging from 10% to 50%. The tube was then fitted to a swinging bucket rotor (SW32) and centrifuged for 5 h at 150,000 × *g*. The OMV band obtained between 20% and 30% sucrose was collected using a long syringe, and the collected OMVs were concentrated by centrifuging at 150,000 × *g* for 2 h. The pellet was resuspended in 500 μL of PBS buffer and filtered through a 0.22-μm syringe filter, and a portion of obtained vesicle suspension (10 μg) was plated on an LB agar plate to check for bacterial contamination. Pure OMVs were then divided into small (50-μL) aliquots and stored at −80°C until further use.

### Transmission electron microscopy.

OMVs (0.5 μg/μL protein concentration) dissolved in PBS were carefully placed on a copper grid (200 mesh), and the excess liquid was removed by gently touching the grid with filter paper. The copper grid was then left in a cool, dry place for 20 min to facilitate the absorption of OMVs. OMVs absorbed on the copper grid were stained by floating the grid in 10 μL aqueous uranyl acetate (2%) for 60 s. The excess stain was removed by gently touching the grid with filter paper. The dried grid was then visualized under a transmission electron microscope (JEOL JEM-1400 electron microscope, JEOL, Ltd.) operating at 120 kV.

### Nanoparticle tracking analysis.

Nanoparticle tracking analysis (NTA) was performed to measure the size distribution of OMVs prepared from A. baumannii DS002. The diluted pure OMVs (0.05 μg/mL) were loaded into the NTA chamber, and the particle size was recorded for 60 s at a laser wavelength of 488 nm using a particle matrix analyser (ZetaView).

### Preparation of subcellular fractions.

An A. baumannii DS002 cell pellet obtained while preparing outer membrane vesicles (OMVs) was used for preparing subcellular fractions (56). Briefly, the flash-frozen cell pellet was washed with phosphate-buffered saline (pH 7.3) before being resuspended (4 mL/g cells) in a 20% sucrose solution prepared using 20 mM Tris EDTA (100 mM) buffer (pH 8.0). The cell suspension was mixed with lysozyme (600 μg/g cells) and the contents were incubated on ice for 40 min. After lysozyme treatment, the appropriate amounts of MgCl_2_ (0.16 mL/g cells) were added from a stock solution and the spheroplasts formed were separated by centrifuging the contents (95,000 × *g*) for 20 min. Supernatant was removed and the pellet containing spheroplasts were resuspended in ice-cold 10 mM Tris HCl (pH 8.0) and sonicated (10 sec on and 30 sec off) for 15 min. Unbroken cells were spun down by centrifugation at 8,000 × *g* for 20 min at 4°C, and the supernatant was centrifuged at 50,000 × *g* for 2 h to pellet the membrane from the cytoplasm. The supernatant contained the cytoplasmic fraction, and the pellet contained the crude membranes. The pellet was washed with 10 mM Tris-HCl (pH 8.0), then resuspended in 1 mL of sterile water. The dissolved membrane was then flash-frozen and thawed by placing it on an ice bucket. The membrane fraction was then adjusted to 0.5% (wt/vol) sarkosyl (sodium-*N*-lauroyl sarcosine) and the contents were incubated at 25°C for 20 min with an end-to-end rotation. The contents were again centrifuged at 50,000 × *g* for 2 h at 4°C. The pellet containing outer membrane (OM) was dissolved in 10 mM Tris-HCl (pH 8.0) and stored at −80°C until further use. The supernatant containing inner membrane was collected into a separate sterile tube.

### Determination of OMV purity.

The purity of OMVs was established by detecting marker proteins. RepA was used as a cytoplasmic protein marker, whereas OmpA was used as an OMV and outer membrane marker protein. The proteins of subcellular fractions prepared from A. baumannii DS002 were separated on 12% SDS-PAGE along with OMV proteins, and independently probed with antibodies of either RepA or OmpA. If RepA-specific signal was absent in the OMV proteins, the OMV preparations were considered pure. The presence of OmpA exclusively in the OMV and outer membrane fractions reconfirmed the purity of the OMVs. Further, the total proteomes of OMVs were searched for the presence of SecYEG, the typical inner membrane-associated protein transport channel, to ascertain the presence of an inner membrane fraction in OMVs.

### Preparation of antibodies.

Preparation of RepA antibodies is described elsewhere (15). Antibodies against OmpA were prepared by immunizing rabbits with recombinant OmpA. Initially, the *ompA* gene was amplified from the genomic DNA of A. baumannii DS002 using a primer set (GD1 FP/GD1 RP) appended with NdeI and XhoI (Table S3). The resulting amplicon was digested with NdeI and XhoI and then cloned in pET28a digested with similar enzymes. The generated recombinant plasmid, pGD1, codes for OmpA^N6XHis^. The OmpA^N6XHis^ was affinity-purified and used for the immunization of rabbits following procedures standardized in our laboratory (15). After immunization, the serum was collected from the immunized rabbits and diluted with an equal volume of equilibration buffer (20 mM sodium phosphate buffer [pH 7.0]). The diluted serum was applied to the protein A column, and the IgGs bound to the column were eluted with elution buffer (0.1 M glycine-HCl [pH 2.7]). The eluted IgGs were then stored at −20°C in 100-μL aliquots until further use. When necessary, appropriate amounts of antibody solution were added to the blot, containing subcellular fractions and OMV proteins of A. baumannii. Western blotting was performed following standard procedures (54).

### Identification of OMV-associated proteins.

Proteomic analysis was performed on biological replicate samples. The purified OMVs were resuspended in 2 × SDS-PAGE sample buffer and boiled for five min at 100°C. A sample corresponding to 30 μg (total protein concentration) was separated on 12% SDS-PAGE and the gels were stained with Coomassie brilliant blue R-250. The lane containing OMV proteins was divided into five zones and cut into separate pieces, and the proteins found in each gel piece were subjected to in-gel digestion with trypsin using standard protocols, as described earlier (57). Briefly, the gel pieces were washed with water, followed by washing with 50 mM ammonium bicarbonate and acetonitrile (1:1) until the stain was removed; later, the gel pieces were treated with acetonitrile (ACN) and dried. They were then incubated with trypsin (10 ng/mL) at 37°C for 16 to 18 h. The in-gel digested peptides were extracted with 30% acetonitrile in water containing 0.1% triflouroacetic acid (TFA) and concentrated with the help of a speed vac concentrator. The peptides were desalted after being dissolved in 5% acetonitrile containing 0.1% TFA using Ziptips, and the sample was analyzed using LC-MS/MS.

### MALDI TOF/TOF of some protein bands.

The peptides generated from the *in-gel* digestion were used to spot on the MALDI target plate. Initially, 2 μL of the peptide sample was spotted and allowed to dry before spotting 2 μL of HCCA matrix. The MALDI plate was then inserted into an MS instrument obtained from Bruker Daltonics (Bremen, Germany) and the acquired MS and MS/MS spectra of different peptides. MS data were acquired from 800 to 4,000 *m/z*. Proteins were identified using the MASCOT program.

### Proteome analysis by LC-ESI-MS/MS.

Peptides generated after trypsin digestion were subjected to LC-ESI-MS/MS using a Q Exactive mass spectrometer obtained from Thermo Fisher Scientific. The peptides were separated on a PepMap RSLC C18 nanocolumn with a pore size of 100 Å and a particle size of 3 μm (Thermo Fisher Scientific), at a flow rate of 300 nL/min, on a 60 min gradient. A liquid chromatography system was connected to ESI-MS/MS, which recorded the collision-induced dissociation (CID) MS of the peptides. The mass resolution of the precursor ion scans was 70,000. The mobile phases A and B were 0.1% formic acid in 5% ACN and 0.1% formic acid in 95% ACN, respectively. The gradient used for separating peptides is shown in Table S4. The MS/MS spectrum of the top 10 peptides with signal threshold of 500 counts was acquired with a 30-sec activation time and a 30-sec repeat duration. The MS/MS data were analyzed using data of Acinetobacter baumannii DS002 from NCBI, accession no. CP027704.1, using Proteome Discoverer version 2.2. The precursor ion mass accuracy was set at 5 ppm and the fragment ion mass accuracy was set at 0.05 Da for the identification of proteins. Additionally, variable modification of methionine oxidation and fixed modification of carbamidomethyl were used for the identification of proteins. Two missed cleavages were allowed for trypsin, and peptides with high confidence were selected. The proteomics data identified in this study were submitted to ProteomeExchange via PRIDE with the identifier PXD026751 (58).

### Functional annotation of identified proteins.

Functions of the identified proteins from the OMVs were predicted using eggNOG-mapper tool version 5.0 (http://eggnog-mapper.embl.de/) (59). The FASTA sequence file was uploaded with the default search filter, and the taxonomic scope selected as gamma-proteobacteria. Results were obtained in Excel format, where the COG category was mentioned for successfully annotated proteins.

### Bioinformatic analysis of the OMV proteins.

The Acinetobacter baumannii DS002 genome-coded proteins were downloaded in a FASTA format from the NCBI database (accession no. CP027704.1). The protein subcellular localization was predicted using Psortb version 3.0 (https://www.psort.org/psortb/) (60). A score of 7 out of 10 was used to predict the localization of proteins with reasonable confidence. If this tool failed to predict localization, such proteins were further analyzed using the program CELLO2GO (http://cello.life.nctu.edu.tw/cello2go/) (61).

### OMVs in iron uptake.

**(i) Preparation of radiolabeled ferric-enterobactin.** Radiolabeled iron-enterobactin complexes were prepared and purified using the following procedure, optimized by our laboratory (62). Initially, a stock solution of desferri-enterobactin (Sigma-Aldrich, USA) was prepared by dissolving 1 mg of desferri-enterobactin in 100 μL of dimethyl sulfoxide. About 3 μL of enterobactin stock solution was taken in a sterile Eppendorf tube, and 5 μL of ^55^Fe was added from 0.2 μmol of ^55^Fe stock (American Radiolabeled Chemicals, MO, USA) (specific activity, 44.6 mCi/mg) and incubated at room temperature for 5 min. The contents were made up to 50 μL with PBS buffer (pH 7.3) and unbound ^55^Fe was removed by passing the reaction mix through a Sephadex G-25 column. The radiolabeled ferric-enterobactin eluted in the flowthrough was collected, and radioactivity was determined by taking 2 μL of ^55^Fe-Ent into 5 mL of scintillation fluid [2,5-diphenyloxazole and 1, 4-bis(5-phenyl-2-oxazolyl) benzene]. The amount of radioactivity found in purified ^55^Fe-Ent was measured using a Perkin Elmer Tri-Carb 2910TR scintillation counter.

**(ii) Labeling of OMVs with ^55^Fe-Ent.** The ^55^Fe-Ent (25 μL/166 pmol) was incubated with 200 μL of OMV (400 μg protein) and the mixture was then incubated at 4°C for 2 h with end-to-end rotation. After incubation, the OMVs were repurified by centrifuging the contents at 150,000 × *g* for 2 h at 4°C. The supernatant was carefully removed, and the pelleted OMVs were redissolved and repurified by repeating the process until negligible counts were seen in the supernatant. The labeled OMVs were then resuspended in 50 μL PBS (pH 7.3) and the amount of ^55^Fe-Ent bound to OMVs was determined by measuring the radioactivity. The labeled OMVs were then stored at −80°C until further use.

**(iii) Iron uptake studies.** Initially, the A. baumannii DS002 cells were acclimatized to grow in minimal medium by growing them in iron sufficient (0.2 μg/mL) minimal medium. Once the culture OD_600_ reached 0.7, the cells were harvested and reinoculated (OD_600_ 0.05) in iron-limiting (0.002 μg/mL) minimal medium and allowed to grow at 30°C until the OD_600_ reached 0.5. The cells were then harvested and washed twice with iron-free minimal salt medium. The cell pellet was then resuspended in iron-limiting medium to obtain a cell suspension of 1.0 OD_600_. The cell suspension (100 μL) was incubated with ^55^Fe-Ent labeled OMV (equivalent to 12 pmol of ^55^Fe). Cells incubated with unlabeled OMVs served as a negative control. These cell suspensions were incubated overnight at 30°C to facilitate iron uptake. Cells were harvested from both the control and experimental samples and extensively washed (twice with 0.1 M LiCl_2_, then once with cold iron-free minimal medium) to remove surface-bound ^55^Fe-Ent from the cells. The washed cell pellets were resuspended in 100 μL minimal media and the associated radioactivity was determined by pipetting 2 μL of the cells into 5 mL scintillation fluid.
