# Supplementary material for: Outer Membrane Vesicles of Acinetobacter baumannii DS002 Are Selectively Enriched with TonB-Dependent Transporters and Play a Key Role in Iron Acquisition
Source: Microbiol Spectr. 2022 Mar 10;10(2):e00293-22. doi: 10.1128/spectrum.00293-22 (PMC9045253; doi:10.1128/spectrum.00293-22)
Supplement: SUPPLEMENTAL FILE 1 — Supplemental material. Download SPECTRUM00293-22_Supp_1_seq8.pdf, PDF file, 0.3 MB [file spectrum00293-22_supp_1_seq8.pdf]

Table S1: List of 302 proteins with their putative functions.

Acc. No.	Protein Name	Description by eggNOG Mapper	COG_category	FUNCTION CLASS	FUNCTION
AVP32905.1	Outer-membrane lipoprotein carrier protein	Participates in the translocation of lipoproteins from the inner membrane to the outer membrane. Only forms a complex with a lipoprotein if the residue after the N-term	M	CELLULAR PROCESSES AND SIGNALING	Cell wall/membrane/envelope biogenesis (M)
AVP32912.1	Multidrug efflux pump subunit AcrA	Belongs to the membrane fusion protein (MFP) (TC 8.A.1) family	M	CELLULAR PROCESSES AND SIGNALING	Cell wall/membrane/envelope biogenesis (M)
AVP32993.1	Outer membrane protein Omp38	Belongs to the ompA family	M	CELLULAR PROCESSES AND SIGNALING	Cell wall/membrane/envelope biogenesis (M)
AVP33066.1	Photosystem I P700 chlorophyll a apoprotein A2	Belongs to the ompA family	M	CELLULAR PROCESSES AND SIGNALING	Cell wall/membrane/envelope biogenesis (M)
AVP33362.1	hypothetical protein C6W84_03545	Nucleoside-specific channel-forming protein, Tsx	M	CELLULAR PROCESSES AND SIGNALING	Cell wall/membrane/envelope biogenesis (M)
AVP33433.1	L-Ala--D-Glu endopeptidase	Peptidase family M23	M	CELLULAR PROCESSES AND SIGNALING	Cell wall/membrane/envelope biogenesis (M)
AVP33493.1	Membrane-bound lytic murein transglycosylase B	Lytic murein transglycosylase	M	CELLULAR PROCESSES AND SIGNALING	Cell wall/membrane/envelope biogenesis (M)
AVP33501.1	Peptidyl-prolyl cis-trans isomerase A	PPIases accelerate the folding of proteins. It catalyzes the cis-trans isomerization of proline imidic peptide bonds in oligopeptides	M	CELLULAR PROCESSES AND SIGNALING	Cell wall/membrane/envelope biogenesis (M)
AVP33616.1	Vitamin B12 transporter BtuB	TonB dependent receptor	M	CELLULAR PROCESSES AND SIGNALING	Cell wall/membrane/envelope biogenesis (M)
AVP33651.1	D-alanyl-D-alanine endopeptidase	Belongs to the peptidase S11 family	M	CELLULAR PROCESSES AND SIGNALING	Cell wall/membrane/envelope biogenesis (M)
AVP33863.1	Tail-specific protease	Belongs to the peptidase S41A family	M	CELLULAR PROCESSES AND SIGNALING	Cell wall/membrane/envelope biogenesis (M)
AVP33874.1	Outer membrane protein assembly factor BamB	Part of the outer membrane protein assembly complex, which is involved in assembly and insertion of beta-barrel proteins into the outer membrane	M	CELLULAR PROCESSES AND SIGNALING	Cell wall/membrane/envelope biogenesis (M)
AVP33882.1	Toxin and drug export protein A	Outer membrane efflux protein	M	CELLULAR PROCESSES AND SIGNALING	Cell wall/membrane/envelope biogenesis (M)
AVP33884.1	LPS-assembly lipoprotein LptE	Together with LptD, is involved in the assembly of lipopolysaccharide (LPS) at the surface of the outer membrane. Required for the proper assembly of LptD. Binds LPS and	M	CELLULAR PROCESSES AND SIGNALING	Cell wall/membrane/envelope biogenesis (M)
AVP34056.1	Outer-membrane lipoprotein LolB	Plays a critical role in the incorporation of lipoproteins in the outer membrane after they are released by the LolA protein	M	CELLULAR PROCESSES AND SIGNALING	Cell wall/membrane/envelope biogenesis (M)
AVP34059.1	Outer membrane protein assembly factor BamD	Part of the outer membrane protein assembly complex, which is involved in assembly and insertion of beta-barrel proteins into the outer membrane	M	CELLULAR PROCESSES AND SIGNALING	Cell wall/membrane/envelope biogenesis (M)
AVP34094.1	putative lipoprotein YiaD	Belongs to the ompA family	M	CELLULAR PROCESSES AND SIGNALING	Cell wall/membrane/envelope biogenesis (M)
AVP34115.1	Gamma-glutamyltranspeptidase	Gamma-glutamyltranspeptidase	M	CELLULAR PROCESSES AND SIGNALING	Cell wall/membrane/envelope biogenesis (M)
AVP34209.1	Membrane-bound lytic murein transglycosylase D	COG0741 Soluble lytic murein transglycosylase and related regulatory proteins (some contain LysM invasin domains)	M	CELLULAR PROCESSES AND SIGNALING	Cell wall/membrane/envelope biogenesis (M)
AVP34252.1	Putative lipoprotein	Curli production assembly/transport component CsgG	M	CELLULAR PROCESSES AND SIGNALING	Cell wall/membrane/envelope biogenesis (M)
AVP34296.1	Metalloprotease LoiP	Peptidase family M48	M	CELLULAR PROCESSES AND SIGNALING	Cell wall/membrane/envelope biogenesis (M)
AVP34558.1	hypothetical protein C6W84_11740	Outer membrane protein (OmpH-like)	M	CELLULAR PROCESSES AND SIGNALING	Cell wall/membrane/envelope biogenesis (M)
AVP34598.1	Putative phospholipase A1	Phospholipase	M	CELLULAR PROCESSES AND SIGNALING	Cell wall/membrane/envelope biogenesis (M)
AVP34773.1	Major outer membrane protein P.IB	Gram-negative porin	M	CELLULAR PROCESSES AND SIGNALING	Cell wall/membrane/envelope biogenesis (M)
AVP34862.1	Chaperone SurA	Chaperone involved in the correct folding and assembly of outer membrane proteins. Recognizes specific patterns of aromatic residues and the orientation of their side c	M	CELLULAR PROCESSES AND SIGNALING	Cell wall/membrane/envelope biogenesis (M)
AVP34890.1	Beta-lactamase OXA-133	Penicillin binding protein transpeptidase domain	M	CELLULAR PROCESSES AND SIGNALING	Cell wall/membrane/envelope biogenesis (M)
AVP35121.1	Membrane-bound lytic murein transglycosylase B	Transglycosylase SLT domain	M	CELLULAR PROCESSES AND SIGNALING	Cell wall/membrane/envelope biogenesis (M)
AVP35122.1	Endolytic peptidoglycan transglycosylase RlpA	Lytic transglycosylase with a strong preference for naked glycan strands that lack stem peptides	M	CELLULAR PROCESSES AND SIGNALING	Cell wall/membrane/envelope biogenesis (M)
AVP35163.1	Putative L,D-transpeptidase YkuD	ErfK YbiS YcfS YnhG family protein	M	CELLULAR PROCESSES AND SIGNALING	Cell wall/membrane/envelope biogenesis (M)
AVP35285.1	Peptidoglycan-associated lipoprotein	Belongs to the ompA family	M	CELLULAR PROCESSES AND SIGNALING	Cell wall/membrane/envelope biogenesis (M)
AXU43350.1	Antibiotic efflux pump outer membrane protein ArpC	Outer membrane efflux protein	M	CELLULAR PROCESSES AND SIGNALING	Cell wall/membrane/envelope biogenesis (M)
AXU43351.1	Solvent efflux pump outer membrane protein SrpC	Outer membrane efflux protein	M	CELLULAR PROCESSES AND SIGNALING	Cell wall/membrane/envelope biogenesis (M)
AXU43387.1	Porin B	Carbohydrate-selective porin, OprB family	M	CELLULAR PROCESSES AND SIGNALING	Cell wall/membrane/envelope biogenesis (M)
AXU43427.1	AXU43427.1	COG0741 Soluble lytic murein transglycosylase and related regulatory proteins (some contain LysM invasin domains)	M	CELLULAR PROCESSES AND SIGNALING	Cell wall/membrane/envelope biogenesis (M)
AXU43632.1	hypothetical protein C6W84_1565	Has lipid A 3-O-deacylase activity. Hydrolyzes the ester bond at the 3 position of lipid A, a bioactive component of lipopolysaccharide (LPS), thereby releasing the primary	M	CELLULAR PROCESSES AND SIGNALING	Cell wall/membrane/envelope biogenesis (M)
AXU43670.1	putative FKBP-type peptidyl-prolyl cis-trans isomerase FkpA	peptidyl-prolyl cis-trans isomerase	M	CELLULAR PROCESSES AND SIGNALING	Cell wall/membrane/envelope biogenesis (M)
AXU43672.1	Polysialic acid transport protein KpsD	Polysaccharide biosynthesis/export protein	M	CELLULAR PROCESSES AND SIGNALING	Cell wall/membrane/envelope biogenesis (M)
AXU43713.1	Soluble lytic murein transglycosylase	Transglycosylase SLT domain	M	CELLULAR PROCESSES AND SIGNALING	Cell wall/membrane/envelope biogenesis (M)
AXU43751.1	hypothetical protein C6W84_2160	OmpW family	M	CELLULAR PROCESSES AND SIGNALING	Cell wall/membrane/envelope biogenesis (M)
AXU43838.1	hypothetical protein C6W84_2595	OmpA family	M	CELLULAR PROCESSES AND SIGNALING	Cell wall/membrane/envelope biogenesis (M)
AXU43869.1	putative phospholipid-binding lipoprotein MlaA	MlaA lipoprotein	M	CELLULAR PROCESSES AND SIGNALING	Cell wall/membrane/envelope biogenesis (M)
AXU44210.1	Outer membrane protein assembly factor BamA	Part of the outer membrane protein assembly complex, which is involved in assembly and insertion of beta-barrel proteins into the outer membrane	M	CELLULAR PROCESSES AND SIGNALING	Cell wall/membrane/envelope biogenesis (M)
AXU44211.1	Outer membrane protein assembly factor BamA	Part of the outer membrane protein assembly complex, which is involved in assembly and insertion of beta-barrel proteins into the outer membrane	M	CELLULAR PROCESSES AND SIGNALING	Cell wall/membrane/envelope biogenesis (M)
AXU44499.1	hypothetical protein C6W84_5900	OmpW family	M	CELLULAR PROCESSES AND SIGNALING	Cell wall/membrane/envelope biogenesis (M)
AXU44500.1	hypothetical protein C6W84_5905	OmpW family	M	CELLULAR PROCESSES AND SIGNALING	Cell wall/membrane/envelope biogenesis (M)
AXU43614.1	hypothetical protein C6W84_1475	Gram-negative-bacterium-type cell outer membrane assembly	M	CELLULAR PROCESSES AND SIGNALING	Cell wall/membrane/envelope biogenesis (M)
AXU44343.1	LPS-assembly protein LptD	lipopolysaccharide transport	M	CELLULAR PROCESSES AND SIGNALING	Cell wall/membrane/envelope biogenesis (M)
AXU43372.1	putative NTE family protein	Esterase of the alpha-beta hydrolase superfamily	M	CELLULAR PROCESSES AND SIGNALING	Cell wall/membrane/envelope biogenesis (M)
AVP35179.1	D-alanyl-D-alanine carboxypeptidase DacA	Belongs to the peptidase S11 family	M	CELLULAR PROCESSES AND SIGNALING	Cell wall/membrane/envelope biogenesis (M)
AVP34631.1	Porin B	wide pore channel activity	M	CELLULAR PROCESSES AND SIGNALING	Cell wall/membrane/envelope biogenesis (M)
AVP35300.1	hypothetical protein C6W84_16625	Outer membrane efflux protein	M	CELLULAR PROCESSES AND SIGNALING	Cell wall/membrane/envelope biogenesis (M)
AVP34177.1	Lipoprotein NlpE	regulation of cell-substrate adhesion	M	CELLULAR PROCESSES AND SIGNALING	Cell wall/membrane/envelope biogenesis (M)
AVP32981.1	putative thiol:disulfide interchange protein DsbC	Required for disulfide bond formation in some periplasmic proteins. Acts by transferring its disulfide bond to other proteins and is reduced in the process	O	CELLULAR PROCESSES AND SIGNALING	Post-translational modification, protein turnover, and chaperones (O)
AVP33025.1	hypothetical protein C6W84_01290	META domain	O	CELLULAR PROCESSES AND SIGNALING	Post-translational modification, protein turnover, and chaperones (O)
AVP33520.1	Thiol:disulfide interchange protein DsbA	Thiol disulfide interchange protein	O	CELLULAR PROCESSES AND SIGNALING	Post-translational modification, protein turnover, and chaperones (O)
AXU44584.1	Periplasmic pH-dependent serine endoprotease DegQ	Belongs to the peptidase S1C family	O	CELLULAR PROCESSES AND SIGNALING	Post-translational modification, protein turnover, and chaperones (O)
AVP35272.1	hypothetical protein C6W84_16425	protein with SCP PR1 domains	O	CELLULAR PROCESSES AND SIGNALING	Post-translational modification, protein turnover, and chaperones (O)
AVP34606.1	ATP-dependent Clp protease ATP-binding subunit ClpA	Belongs to the ClpA ClpB family	O	CELLULAR PROCESSES AND SIGNALING	Post-translational modification, protein turnover, and chaperones (O)
AVP34461.1	Peptidyl-prolyl cis-trans isomerase cyp18	PPIases accelerate the folding of proteins. It catalyzes the cis-trans isomerization of proline imidic peptide bonds in oligopeptides	O	CELLULAR PROCESSES AND SIGNALING	Post-translational modification, protein turnover, and chaperones (O)
AVP34317.1	Alkyl hydroperoxide reductase subunit C	alkyl hydroperoxide reductase	O	CELLULAR PROCESSES AND SIGNALING	Post-translational modification, protein turnover, and chaperones (O)
AVP33947.1	Thioredoxin C-2	Belongs to the thioredoxin family	O	CELLULAR PROCESSES AND SIGNALING	Post-translational modification, protein turnover, and chaperones (O)
AVP33530.1	putative FKBP-type peptidyl-prolyl cis-trans isomerase FkpA	Peptidyl-prolyl cis-trans isomerase	O	CELLULAR PROCESSES AND SIGNALING	Post-translational modification, protein turnover, and chaperones (O)
AVP32929.1	Modulator of FtsH protease HflK	stress-induced mitochondrial fusion	O	CELLULAR PROCESSES AND SIGNALING	Post-translational modification, protein turnover, and chaperones (O)
AVP34707.1	Biopolymer transport protein ExbD	Biopolymer transport protein ExbD/TolR	U	CELLULAR PROCESSES AND SIGNALING	Intracellular trafficking, secretion, and vesicular transport (U)
AVP34963.1	hypothetical protein C6W84_14240	hemolysin activation secretion protein	U	CELLULAR PROCESSES AND SIGNALING	Intracellular trafficking, secretion, and vesicular transport (U)
AVP35284.1	Protein TolB	Involved in the TonB-independent uptake of proteins	U	CELLULAR PROCESSES AND SIGNALING	Intracellular trafficking, secretion, and vesicular transport (U)
AXU43834.1	hypothetical protein C6W84_2575	TIGRFAM conserved repeat domain	U	CELLULAR PROCESSES AND SIGNALING	Intracellular trafficking, secretion, and vesicular transport (U)
AVP35252.1	Signal peptidase I	signal peptide processing	U	CELLULAR PROCESSES AND SIGNALING	Intracellular trafficking, secretion, and vesicular transport (U)
AVP33228.1	Type IV pilus biogenesis and competence protein PilQ	Type ii and iii secretion system protein	U	CELLULAR PROCESSES AND SIGNALING	Intracellular trafficking, secretion, and vesicular transport (U)
AXU44519.1	Beta-lactamase	Beta-lactamase	V	CELLULAR PROCESSES AND SIGNALING	Defense mechanisms (V)
AXU44520.1	Beta-lactamase	Beta-lactamase	V	CELLULAR PROCESSES AND SIGNALING	Defense mechanisms (V)
AVP34562.1	Cell division coordinator CpoB	TolA binding protein trimerisation	D	CELLULAR PROCESSES AND SIGNALING	Cell cycle control, cell division, chromosome partitioning (D)
AXU43953.1	Septum site-determining protein MinD	cell division	D	CELLULAR PROCESSES AND SIGNALING	Cell cycle control, cell division, chromosome partitioning (D)
AVP33531.1	Tyrosine-protein kinase ptk	protein tyrosine kinase activity	D	CELLULAR PROCESSES AND SIGNALING	Cell cycle control, cell division, chromosome partitioning (D)
AVP34373.1	hypothetical protein C6W84_10445	Universal stress protein	T	CELLULAR PROCESSES AND SIGNALING	Signal transduction mechanisms (T)
AVP33997.1	Transcriptional regulatory protein RstA	Transcriptional regulatory protein, C terminal	T	CELLULAR PROCESSES AND SIGNALING	Signal transduction mechanisms (T)
AVP33077.1	50S ribosomal protein L13	This protein is one of the early assembly proteins of the 50S ribosomal subunit, although it is not seen to bind rRNA by itself. It is important during the early stages of 50S	J	INFORMATION STORAGE AND PROCESSING	Translation, ribosomal structure and biogenesis (J)
AVP33078.1	30S ribosomal protein S9	Belongs to the universal ribosomal protein uS9 family	J	INFORMATION STORAGE AND PROCESSING	Translation, ribosomal structure and biogenesis (J)
AVP33090.1	hypothetical protein C6W84_01765	Belongs to the RNase T2 family	J	INFORMATION STORAGE AND PROCESSING	Translation, ribosomal structure and biogenesis (J)
AVP33112.1	50S ribosomal protein L17	Ribosomal protein L17	J	INFORMATION STORAGE AND PROCESSING	Translation, ribosomal structure and biogenesis (J)
AVP33114.1	30S ribosomal protein S4	One of the primary rRNA binding proteins, it binds directly to 16S rRNA where it nucleates assembly of the body of the 30S subunit	J	INFORMATION STORAGE AND PROCESSING	Translation, ribosomal structure and biogenesis (J)
AVP33115.1	30S ribosomal protein S11	Located on the platform of the 30S subunit, it bridges several disparate RNA helices of the 16S rRNA. Forms part of the Shine-Dalgarno cleft in the 70S ribosome	J	INFORMATION STORAGE AND PROCESSING	Translation, ribosomal structure and biogenesis (J)
AVP33119.1	50S ribosomal protein L15	binds to the 23S rRNA	J	INFORMATION STORAGE AND PROCESSING	Translation, ribosomal structure and biogenesis (J)
AVP33122.1	50S ribosomal protein L18	This is one of the proteins that binds and probably mediates the attachment of the 5S RNA into the large ribosomal subunit, where it forms part of the central protuberar	J	INFORMATION STORAGE AND PROCESSING	Translation, ribosomal structure and biogenesis (J)
AVP33123.1	50S ribosomal protein L6	This protein binds to the 23S rRNA, and is important in its secondary structure. It is located near the subunit interface in the base of the L7 L12 stalk, and near the tRNA b	J	INFORMATION STORAGE AND PROCESSING	Translation, ribosomal structure and biogenesis (J)
AVP33124.1	30S ribosomal protein S8	One of the primary rRNA binding proteins, it binds directly to 16S rRNA central domain where it helps coordinate assembly of the platform of the 30S subunit	J	INFORMATION STORAGE AND PROCESSING	Translation, ribosomal structure and biogenesis (J)

AVP33126.1	50S ribosomal protein L5	This is 1 of the proteins that binds and probably mediates the attachment of the 5S RNA into the large ribosomal subunit, where it forms part of the central protuberance	J	INFORMATION STORAGE AND PROCESSING	Translation, ribosomal structure and biogenesis (J)
AVP33128.1	50S ribosomal protein L14	Binds to 23S rRNA. Forms part of two intersubunit bridges in the 70S ribosome	J	INFORMATION STORAGE AND PROCESSING	Translation, ribosomal structure and biogenesis (J)
AVP33131.1	50S ribosomal protein L16	Binds 23S rRNA and is also seen to make contacts with the A and possibly P site tRNAs	J	INFORMATION STORAGE AND PROCESSING	Translation, ribosomal structure and biogenesis (J)
AVP33132.1	30S ribosomal protein S3	Binds the lower part of the 30S subunit head. Binds mRNA in the 70S ribosome, positioning it for translation	J	INFORMATION STORAGE AND PROCESSING	Translation, ribosomal structure and biogenesis (J)
AVP33135.1	50S ribosomal protein L2	One of the primary rRNA binding proteins. Required for association of the 30S and 50S subunits to form the 70S ribosome, for tRNA binding and peptide bond formation.	J	INFORMATION STORAGE AND PROCESSING	Translation, ribosomal structure and biogenesis (J)
AVP33136.1	50S ribosomal protein L23	One of the early assembly proteins it binds 23S rRNA. One of the proteins that surrounds the polypeptide exit tunnel on the outside of the ribosome. Forms the main doc	J	INFORMATION STORAGE AND PROCESSING	Translation, ribosomal structure and biogenesis (J)
AVP33138.1	50S ribosomal protein L3	One of the primary rRNA binding proteins, it binds directly near the 3'-end of the 23S rRNA, where it nucleates assembly of the 50S subunit	J	INFORMATION STORAGE AND PROCESSING	Translation, ribosomal structure and biogenesis (J)
AVP33206.1	50S ribosomal protein L19	This protein is located at the 30S-50S ribosomal subunit interface and may play a role in the structure and function of the aminoacyl-tRNA binding site	J	INFORMATION STORAGE AND PROCESSING	Translation, ribosomal structure and biogenesis (J)
AVP33686.1	50S ribosomal protein L11	Forms part of the ribosomal stalk which helps the ribosome interact with GTP-bound translation factors	J	INFORMATION STORAGE AND PROCESSING	Translation, ribosomal structure and biogenesis (J)
AVP33687.1	50S ribosomal protein L1	Binds directly to 23S rRNA. The L1 stalk is quite mobile in the ribosome, and is involved in E site tRNA release	J	INFORMATION STORAGE AND PROCESSING	Translation, ribosomal structure and biogenesis (J)
AVP34079.1	30S ribosomal protein S7	One of the primary rRNA binding proteins, it binds directly to 16S rRNA where it nucleates assembly of the head domain of the 30S subunit. Is located at the subunit inter	J	INFORMATION STORAGE AND PROCESSING	Translation, ribosomal structure and biogenesis (J)
AVP34081.1	Elongation factor Tu	This protein promotes the GTP-dependent binding of aminoacyl-tRNA to the A-site of ribosomes during protein biosynthesis	J	INFORMATION STORAGE AND PROCESSING	Translation, ribosomal structure and biogenesis (J)
AVP34447.1	hypothetical protein C6W84_10985	Part of the outer membrane protein assembly complex, which is involved in assembly and insertion of beta-barrel proteins into the outer membrane	J	INFORMATION STORAGE AND PROCESSING	Translation, ribosomal structure and biogenesis (J)
AVP35129.1	30S ribosomal protein S2	Belongs to the universal ribosomal protein uS2 family	J	INFORMATION STORAGE AND PROCESSING	Translation, ribosomal structure and biogenesis (J)
AXU43962.1	Outer membrane protein assembly factor BamE	Part of the outer membrane protein assembly complex, which is involved in assembly and insertion of beta-barrel proteins into the outer membrane	J	INFORMATION STORAGE AND PROCESSING	Translation, ribosomal structure and biogenesis (J)
AXU43769.1	30S ribosome-binding factor	rRNA processing	J	INFORMATION STORAGE AND PROCESSING	Translation, ribosomal structure and biogenesis (J)
AXU43566.1	hypothetical protein C6W84_1235	regulation of translation	J	INFORMATION STORAGE AND PROCESSING	Translation, ribosomal structure and biogenesis (J)
AVP35171.1	50S ribosomal protein L31	rRNA binding	J	INFORMATION STORAGE AND PROCESSING	Translation, ribosomal structure and biogenesis (J)
AVP35168.1	Elongation factor P	translation elongation factor activity	J	INFORMATION STORAGE AND PROCESSING	Translation, ribosomal structure and biogenesis (J)
AVP35128.1	Elongation factor Ts	Associates with the EF-Tu.GDP complex and induces the exchange of GDP to GTP. It remains bound to the aminoacyl-tRNA.EF- Tu.GTP complex up to the GTP hydrolysis st	J	INFORMATION STORAGE AND PROCESSING	Translation, ribosomal structure and biogenesis (J)
AVP35062.1	30S ribosomal protein S21	Belongs to the bacterial ribosomal protein bS21 family	J	INFORMATION STORAGE AND PROCESSING	Translation, ribosomal structure and biogenesis (J)
AVP34819.1	30S ribosomal protein S20	rRNA binding	J	INFORMATION STORAGE AND PROCESSING	Translation, ribosomal structure and biogenesis (J)
AVP34553.1	Ribosome-recycling factor	cytoplasmic translational termination	J	INFORMATION STORAGE AND PROCESSING	Translation, ribosomal structure and biogenesis (J)
AVP34407.1	30S ribosomal protein S6	Binds together with S18 to 16S ribosomal RNA	J	INFORMATION STORAGE AND PROCESSING	Translation, ribosomal structure and biogenesis (J)
AVP34405.1	50S ribosomal protein L9	rRNA binding	J	INFORMATION STORAGE AND PROCESSING	Translation, ribosomal structure and biogenesis (J)
AVP34406.1	30S ribosomal protein S18	rRNA binding	J	INFORMATION STORAGE AND PROCESSING	Translation, ribosomal structure and biogenesis (J)
AVP34078.1	30S ribosomal protein S12	rRNA binding	J	INFORMATION STORAGE AND PROCESSING	Translation, ribosomal structure and biogenesis (J)
AVP33936.1	50S ribosomal protein L20	Binds directly to 23S ribosomal RNA and is necessary for the in vitro assembly process of the 50S ribosomal subunit. It is not involved in the protein synthesizing functions	J	INFORMATION STORAGE AND PROCESSING	Translation, ribosomal structure and biogenesis (J)
AVP33755.1	Polyribonucleotide nucleotidyltransferase	polyribonucleotide nucleotidyltransferase activity	J	INFORMATION STORAGE AND PROCESSING	Translation, ribosomal structure and biogenesis (J)
AVP33689.1	50S ribosomal protein L7/L12	mitochondrial gene expression	J	INFORMATION STORAGE AND PROCESSING	Translation, ribosomal structure and biogenesis (J)
AVP33688.1	50S ribosomal protein L10	Forms part of the ribosomal stalk, playing a central role in the interaction of the ribosome with GTP-bound translation factors	J	INFORMATION STORAGE AND PROCESSING	Translation, ribosomal structure and biogenesis (J)
AVP33209.1	30S ribosomal protein S16	mitochondrial translation	J	INFORMATION STORAGE AND PROCESSING	Translation, ribosomal structure and biogenesis (J)
AVP33139.1	30S ribosomal protein S10	cytoplasmic translation	J	INFORMATION STORAGE AND PROCESSING	Translation, ribosomal structure and biogenesis (J)
AVP33137.1	50S ribosomal protein L4	Forms part of the polypeptide exit tunnel	J	INFORMATION STORAGE AND PROCESSING	Translation, ribosomal structure and biogenesis (J)
AVP33134.1	30S ribosomal protein S19	Protein S19 forms a complex with S13 that binds strongly to the 16S ribosomal RNA	J	INFORMATION STORAGE AND PROCESSING	Translation, ribosomal structure and biogenesis (J)
AVP33133.1	50S ribosomal protein L22	The globular domain of the protein is located near the polypeptide exit tunnel on the outside of the subunit, while an extended beta-hairpin is found that lines the wall of	J	INFORMATION STORAGE AND PROCESSING	Translation, ribosomal structure and biogenesis (J)
AVP33127.1	50S ribosomal protein L24	One of the proteins that surrounds the polypeptide exit tunnel on the outside of the subunit	J	INFORMATION STORAGE AND PROCESSING	Translation, ribosomal structure and biogenesis (J)
AVP33125.1	30S ribosomal protein S14	Binds 16S rRNA, required for the assembly of 30S particles and may also be responsible for determining the conformation of the 16S rRNA at the A site	J	INFORMATION STORAGE AND PROCESSING	Translation, ribosomal structure and biogenesis (J)
AVP33121.1	30S ribosomal protein S5	rRNA binding	J	INFORMATION STORAGE AND PROCESSING	Translation, ribosomal structure and biogenesis (J)
AVP33117.1	50S ribosomal protein L36	Belongs to the bacterial ribosomal protein bL36 family	J	INFORMATION STORAGE AND PROCESSING	Translation, ribosomal structure and biogenesis (J)
AVP33116.1	30S ribosomal protein S13	Located at the top of the head of the 30S subunit, it contacts several helices of the 16S rRNA. In the 70S ribosome it contacts the 23S rRNA (bridge B1a) and protein L5 of	J	INFORMATION STORAGE AND PROCESSING	Translation, ribosomal structure and biogenesis (J)
AVP32907.1	50S ribosomal protein L21	This protein binds to 23S rRNA in the presence of protein L20	J	INFORMATION STORAGE AND PROCESSING	Translation, ribosomal structure and biogenesis (J)
AVP32940.1	hypothetical protein C6W84_00745	long-chain fatty acid transport protein	I	INFORMATION STORAGE AND PROCESSING	Lipid transport and metabolism (I)
AVP34156.1	Lipase 1	Alpha beta hydrolase	I	INFORMATION STORAGE AND PROCESSING	Lipid transport and metabolism (I)
AVP33525.1	Non-hemolytic phospholipase C	nuclear-transcribed mRNA catabolic process, deadenylation-dependent decay	K	INFORMATION STORAGE AND PROCESSING	Transcription (K)
AVP33113.1	DNA-directed RNA polymerase subunit alpha	RNA polymerase activity	K	INFORMATION STORAGE AND PROCESSING	Transcription (K)
AVP32848.1	Glycerophosphodiester phosphodiesterase, periplasmic	Glycerophosphoryl diester phosphodiesterase family	C	METABOLISM	Energy production and conversion (C)
AVP32886.1	Succinate dehydrogenase iron-sulfur subunit	succinate dehydrogenase	C	METABOLISM	Energy production and conversion (C)
AVP32887.1	Succinate dehydrogenase flavoprotein subunit	Belongs to the FAD-dependent oxidoreductase 2 family. FRD SDH subfamily	C	METABOLISM	Energy production and conversion (C)
AVP33604.1	ATP synthase subunit beta 1	Produces ATP from ADP in the presence of a proton gradient across the membrane. The catalytic sites are hosted primarily by the beta subunits	C	METABOLISM	Energy production and conversion (C)
AVP35306.1	Electron transfer flavoprotein subunit alpha	Electron transfer flavoprotein	C	METABOLISM	Energy production and conversion (C)
AVP35307.1	Electron transfer flavoprotein subunit beta	electron transfer activity	C	METABOLISM	Energy production and conversion (C)
AVP35144.1	Superoxide dismutase	Destroys radicals which are normally produced within the cells and which are toxic to biological systems	C	METABOLISM	Energy production and conversion (C)
AVP34412.1	Cytochrome bo(3) ubiquinol oxidase subunit 2	oxidoreductase activity, acting on a heme group of donors, oxygen as acceptor	C	METABOLISM	Energy production and conversion (C)
AVP34003.1	NADH-quinone oxidoreductase subunit I	NDH-1 shuttles electrons from NADH, via FMN and iron- sulfur (Fe-S) centers, to quinones in the respiratory chain. The immediate electron acceptor for the enzyme in thi	C	METABOLISM	Energy production and conversion (C)
AVP34001.1	NADH-quinone oxidoreductase subunit G	ATP synthesis coupled electron transport	C	METABOLISM	Energy production and conversion (C)
AVP34000.1	NADH-quinone oxidoreductase subunit F	NDH-1 shuttles electrons from NADH, via FMN and iron- sulfur (Fe-S) centers, to quinones in the respiratory chain	C	METABOLISM	Energy production and conversion (C)
AVP33998.1	NADH-quinone oxidoreductase subunit B	NDH-1 shuttles electrons from NADH, via FMN and iron- sulfur (Fe-S) centers, to quinones in the respiratory chain. The immediate electron acceptor for the enzyme in thi	C	METABOLISM	Energy production and conversion (C)
AVP32883.1	Dihydrolipoyllysine-residue succinyltransferase component of 2-oxoglu	S-acyltransferase activity	C	METABOLISM	Energy production and conversion (C)
AVP32882.1	Dihydrolipoyl dehydrogenase	cell redox homeostasis	C	METABOLISM	Energy production and conversion (C)
AVP32881.1	Succinate--CoA ligase [ADP-forming] subunit beta	Succinyl-CoA synthetase functions in the citric acid cycle (TCA), coupling the hydrolysis of succinyl-CoA to the synthesis of either ATP or GTP and thus represents the only	C	METABOLISM	Energy production and conversion (C)
AVP32880.1	Succinate--CoA ligase [ADP-forming] subunit alpha	Succinyl-CoA synthetase functions in the citric acid cycle (TCA), coupling the hydrolysis of succinyl-CoA to the synthesis of either ATP or GTP and thus represents the only	C	METABOLISM	Energy production and conversion (C)
QDX16480.1	Leu/Ile/Val-binding protein (plasmid)	Receptor family ligand binding region	E	METABOLISM	Amino acid transport and metabolism (E)
QDX16481.1	hypothetical protein C6W84_16901 (plasmid)	Receptor family ligand binding region	E	METABOLISM	Amino acid transport and metabolism (E)
AXU44003.1	Esterase TesA	Lysophospholipase L1 and related esterases	E	METABOLISM	Amino acid transport and metabolism (E)
AVP34673.1	Endonuclease YhcR	5'-nucleotidase, C-terminal domain	F	METABOLISM	Nucleotide transport and metabolism (F)
AXU43517.1	Nuclease	DNA RNA non-specific endonuclease	F	METABOLISM	Nucleotide transport and metabolism (F)
AVP33460.1	hypothetical protein C6W84_04265	TRAP-type C4-dicarboxylate transport system periplasmic component	G	METABOLISM	Carbohydrate transport and metabolism (G)
AVP34135.1	Poly-beta-1,6-N-acetyl-D-glucosamine N-deacetylase	Hypothetical glycosyl hydrolase family 13	G	METABOLISM	Carbohydrate transport and metabolism (G)
AXU44186.1	6-phosphogluconolactonase	Lactonase, 7-bladed beta-propeller	G	METABOLISM	Carbohydrate transport and metabolism (G)
AXU43991.1	Aldose 1-epimerase	converts alpha-aldose to the beta-anomer	G	METABOLISM	Carbohydrate transport and metabolism (G)
AXU43615.1	Aldose sugar dehydrogenase YliI	pyrroloquinoline quinone binding	G	METABOLISM	Carbohydrate transport and metabolism (G)
AVP34386.1	Aldose sugar dehydrogenase YliI	pyrroloquinoline quinone binding	G	METABOLISM	Carbohydrate transport and metabolism (G)
AVP33022.1	Vitamin B12 transporter BtuB	TonB dependent receptor	P	METABOLISM	Inorganic ion transport and metabolism (P)
AVP33193.1	Superoxide dismutase [Cu-Zn]	Destroys radicals which are normally produced within the cells and which are toxic to biological systems	P	METABOLISM	Inorganic ion transport and metabolism (P)
AVP33218.1	Bacterioferritin	Iron-storage protein, whose ferroxidase center binds Fe(2) ions, oxidizes them by dioxygen to Fe(3), and participates in the subsequent Fe(3) oxide mineral core formati	P	METABOLISM	Inorganic ion transport and metabolism (P)
AVP33597.1	High-affinity zinc uptake system protein ZnuA	Zinc-uptake complex component A periplasmic	P	METABOLISM	Inorganic ion transport and metabolism (P)
AVP33656.1	Fe(2+) transporter FeoB	Ferrous iron transport protein B	P	METABOLISM	Inorganic ion transport and metabolism (P)
AVP34155.1	Ferric enterobactin receptor	TonB dependent receptor	P	METABOLISM	Inorganic ion transport and metabolism (P)
AVP34214.1	Ferrichrome-iron receptor	TonB dependent receptor	P	METABOLISM	Inorganic ion transport and metabolism (P)
AVP34241.1	Copper resistance protein B	Copper resistance protein B precursor (CopB)	P	METABOLISM	Inorganic ion transport and metabolism (P)
AVP34247.1	Membrane lipoprotein TpN32	NLPA lipoprotein	P	METABOLISM	Inorganic ion transport and metabolism (P)
AVP34394.1	Membrane lipoprotein TpN32	NLPA lipoprotein	P	METABOLISM	Inorganic ion transport and metabolism (P)
AVP34448.1	hypothetical protein C6W84_10985	Domain of unknown function (DUF4198)	P	METABOLISM	Inorganic ion transport and metabolism (P)
AVP34597.1	putative TonB-dependent receptor BfrD	TonB dependent receptor	P	METABOLISM	Inorganic ion transport and metabolism (P)
AVP34668.1	Molybdate-binding periplasmic protein	Bacterial extracellular solute-binding protein	P	METABOLISM	Inorganic ion transport and metabolism (P)
AVP35155.1	Colicin I receptor	TonB dependent receptor	P	METABOLISM	Inorganic ion transport and metabolism (P)
AVP35190.1	Phosphate-binding protein PstS	PBP superfamily domain	P	METABOLISM	Inorganic ion transport and metabolism (P)
AVP35260.1	Sulfate-binding protein	sulfate ABC transporter	P	METABOLISM	Inorganic ion transport and metabolism (P)

AVP35425.1	Ferric aerobactin receptor	TonB dependent receptor	P	METABOLISM	Inorganic ion transport and metabolism (P)
AXU43383.1	hypothetical protein C6W84_0320	TonB dependent receptor	P	METABOLISM	Inorganic ion transport and metabolism (P)
AXU43384.1	Ferric aerobactin receptor	TonB dependent receptor	P	METABOLISM	Inorganic ion transport and metabolism (P)
AXU43402.1	putative TonB-dependent receptor	TonB-dependent receptor	P	METABOLISM	Inorganic ion transport and metabolism (P)
AXU43592.1	Ferrichrome receptor FcuA	TonB-dependent Receptor Plug Domain	P	METABOLISM	Inorganic ion transport and metabolism (P)
AXU43593.1	Ferrichrome receptor FcuA	TonB-dependent Receptor Plug Domain	P	METABOLISM	Inorganic ion transport and metabolism (P)
AXU43696.1	Ferrichrome-iron receptor	TonB dependent receptor	P	METABOLISM	Inorganic ion transport and metabolism (P)
AXU43811.1	putative TonB-dependent receptor BfrD	TonB dependent receptor	P	METABOLISM	Inorganic ion transport and metabolism (P)
AXU43812.1	putative TonB-dependent receptor BfrD	TonB dependent receptor	P	METABOLISM	Inorganic ion transport and metabolism (P)
AXU44011.1	hypothetical protein C6W84_3460	Capsule assembly protein Wzi	P	METABOLISM	Inorganic ion transport and metabolism (P)
AXU44161.1	FhuE receptor	TonB dependent receptor	P	METABOLISM	Inorganic ion transport and metabolism (P)
AXU44162.1	Ferripyoverdine receptor	receptor	P	METABOLISM	Inorganic ion transport and metabolism (P)
AXU44299.1	FhuE receptor	TonB dependent receptor	P	METABOLISM	Inorganic ion transport and metabolism (P)
AXU44326.1	Vitamin B12 transporter BtuB	TonB dependent receptor	P	METABOLISM	Inorganic ion transport and metabolism (P)
QDX16513.1	Fe(3+) dicitrate transport protein FecA (plasmid)	TonB dependent receptor	P	METABOLISM	Inorganic ion transport and metabolism (P)
QDX16557.1	Ferrichrome-iron receptor (plasmid)	TonB dependent receptor	P	METABOLISM	Inorganic ion transport and metabolism (P)
AVP34153.1	Arylsulfatase	Arylsulfatase A and related enzymes	P	METABOLISM	Inorganic ion transport and metabolism (P)
AXU43452.1	putative phospholipid-binding protein MlaC	MlaC protein	Q	METABOLISM	Secondary metabolites biosynthesis, transport, and catabolism (Q)
AXU44046.1	Copper resistance protein A	Multicopper oxidase	Q	METABOLISM	Secondary metabolites biosynthesis, transport, and catabolism (Q)
AVP35097.1	hypothetical protein C6W84_15235	COG0457 FOG TPR repeat	H	METABOLISM	Coenzyme transport and metabolism (H)
AVP32927.1	putative NTE family protein	Patatin-like phospholipase	S	POORLY CHARACTERIZED	Function unknown (S)
AVP32953.1	hypothetical protein C6W84_00820	Protein of unknown function (DUF1176)	S	POORLY CHARACTERIZED	Function unknown (S)
AVP32954.1	hypothetical protein C6W84_00825	Domain of unknown function (DUF4850)	S	POORLY CHARACTERIZED	Function unknown (S)
AVP32982.1	Secretory immunoglobulin A-binding protein EsiB	Sel1-like repeats.	S	POORLY CHARACTERIZED	Function unknown (S)
AVP33073.1	hypothetical protein C6W84_01640	Uncharacterized protein conserved in bacteria (DUF2147)	S	POORLY CHARACTERIZED	Function unknown (S)
AVP33095.1	hypothetical protein C6W84_01800	Protein of unknown function (DUF3108)	S	POORLY CHARACTERIZED	Function unknown (S)
AVP33169.1	hypothetical protein C6W84_02255	Lysozyme inhibitor LprI	S	POORLY CHARACTERIZED	Function unknown (S)
AVP33311.1	hypothetical protein C6W84_03245	Putative general bacterial porin	S	POORLY CHARACTERIZED	Function unknown (S)
AVP33352.1	hypothetical protein C6W84_03490	Uncharacterized protein conserved in bacteria (DUF2147)	S	POORLY CHARACTERIZED	Function unknown (S)
AVP33356.1	hypothetical protein C6W84_03510	Putative MetA-pathway of phenol degradation	S	POORLY CHARACTERIZED	Function unknown (S)
AVP33613.1	hypothetical protein C6W84_05275	LysM domain	S	POORLY CHARACTERIZED	Function unknown (S)
AVP33624.1	hypothetical protein C6W84_05365	Esterase-like activity of phytase	S	POORLY CHARACTERIZED	Function unknown (S)
AVP33637.1	Porin D	outer membrane porin, OprD family	S	POORLY CHARACTERIZED	Function unknown (S)
AVP33638.1	hypothetical protein C6W84_05455	Bacterial protein of unknown function (Gcw_chp)	S	POORLY CHARACTERIZED	Function unknown (S)
AVP33837.1	hypothetical protein C6W84_06755	Bacterial protein of unknown function (DUF839)	S	POORLY CHARACTERIZED	Function unknown (S)
AVP33838.1	hypothetical protein C6W84_06760	Bacterial protein of unknown function (DUF839)	S	POORLY CHARACTERIZED	Function unknown (S)
AVP33844.1	Secretory immunoglobulin A-binding protein EsiB	Sel1-like repeats.	S	POORLY CHARACTERIZED	Function unknown (S)
AVP33891.1	hypothetical protein C6W84_07095	TIGRFAM conserved repeat domain	S	POORLY CHARACTERIZED	Function unknown (S)
AVP34205.1	hypothetical protein C6W84_09380	BON domain	S	POORLY CHARACTERIZED	Function unknown (S)
AVP34250.1	Putative lipoprotein	Putative bacterial lipoprotein (DUF799)	S	POORLY CHARACTERIZED	Function unknown (S)
AVP34251.1	hypothetical protein C6W84_09710	Domain of unknown function (DUF4810)	S	POORLY CHARACTERIZED	Function unknown (S)
AVP34383.1	hypothetical protein C6W84_10550	ABC-type transport auxiliary lipoprotein component	S	POORLY CHARACTERIZED	Function unknown (S)
AVP34662.1	hypothetical protein C6W84_12355	Bacterial protein of unknown function (Gcw_chp)	S	POORLY CHARACTERIZED	Function unknown (S)
AVP34766.1	hypothetical protein C6W84_13005	TonB C terminal	S	POORLY CHARACTERIZED	Function unknown (S)
AVP34785.1	hypothetical protein C6W84_13125	Histidine phosphatase superfamily (branch 2)	S	POORLY CHARACTERIZED	Function unknown (S)
AVP34883.1	Protein YceI	YceI-like domain	S	POORLY CHARACTERIZED	Function unknown (S)
AVP34945.1	hypothetical protein C6W84_14120	Tetratricopeptide repeat	S	POORLY CHARACTERIZED	Function unknown (S)
AVP35115.1	hypothetical protein C6W84_15360	Secretory lipase	S	POORLY CHARACTERIZED	Function unknown (S)
AVP35195.1	hypothetical protein C6W84_15930	Protein of unknown function (DUF541)	S	POORLY CHARACTERIZED	Function unknown (S)
AVP35212.1	putative zinc protease	Belongs to the peptidase M16 family	S	POORLY CHARACTERIZED	Function unknown (S)
AVP35370.1	hypothetical protein C6W84_07080	Domain of unknown function DUF11	S	POORLY CHARACTERIZED	Function unknown (S)
AVP35404.1	hypothetical protein C6W84_10920	Protein of unknown function (DUF2799)	S	POORLY CHARACTERIZED	Function unknown (S)
AXU43373.1	hypothetical protein C6W84_0270	Entericidin EcnA/B family	S	POORLY CHARACTERIZED	Function unknown (S)
AXU43553.1	hypothetical protein C6W84_1170	Putative general bacterial porin	S	POORLY CHARACTERIZED	Function unknown (S)
AXU43836.1	hypothetical protein C6W84_2585	cell adhesion involved in biofilm formation	S	POORLY CHARACTERIZED	Function unknown (S)
AXU44089.1	hypothetical protein C6W84_3850	Peptidase M15	S	POORLY CHARACTERIZED	Function unknown (S)
AXU44481.1	Ferri-bacillibactin esterase BesA	Putative esterase	S	POORLY CHARACTERIZED	Function unknown (S)
QDX16474.1	Porin-like protein NicP (plasmid)	outer membrane porin, OprD family	S	POORLY CHARACTERIZED	Function unknown (S)
QDX16497.1	hypothetical protein C6W84_16917 (plasmid)	Spore Coat Protein U domain	S	POORLY CHARACTERIZED	Function unknown (S)
QDX16498.1	hypothetical protein C6W84_16918 (plasmid)	Spore Coat Protein U domain	S	POORLY CHARACTERIZED	Function unknown (S)
QDX16499.1	hypothetical protein C6W84_16919 (plasmid)	Spore Coat Protein U domain	S	POORLY CHARACTERIZED	Function unknown (S)
QDX16502.1	F1 capsule-anchoring protein (plasmid)	Outer membrane usher protein	S	POORLY CHARACTERIZED	Function unknown (S)
AXU43623.1	hypothetical protein C6W84_1520	START domain	S	POORLY CHARACTERIZED	Function unknown (S)
AVP34588.1	hypothetical protein C6W84_11915	Uncharacterized protein conserved in bacteria (DUF2057)	S	POORLY CHARACTERIZED	Function unknown (S)
AVP34366.1	Lipopolysaccharide export system protein LptA	lipopolysaccharide binding	S	POORLY CHARACTERIZED	Function unknown (S)
AVP33805.1	hypothetical protein C6W84_06540	Lysozyme inhibitor LprI	S	POORLY CHARACTERIZED	Function unknown (S)
AVP35175.1	Murein hydrolase activator NlpD	Peptidase family M23	DM		
AVP34941.1	Glutaminase-asparaginase	Asparaginase	EJ		
AVP34920.1	Glutamate/aspartate import solute-binding protein	Bacterial periplasmic substrate-binding proteins	ET		
AVP35112.1	Lysine/arginine/ornithine-binding periplasmic protein	Bacterial periplasmic substrate-binding proteins	ET		
AXU43664.1	putative oxidoreductase YciK	KR domain	IQ		
AVP32926.1	hypothetical protein C6W84_00650	Forms passive diffusion pores that allow small molecular weight hydrophilic materials across the outer membrane	MU		
AXU43745.1	Outer membrane protein TolC	Outer membrane efflux protein	MU		
AVP33220.1	Fimbrial protein	Belongs to the N-Me-Phe pilin family	NU		
AVP35185.1	hypothetical protein C6W84_15855	Domain of unknown function	NU		
AXU43819.1	Lipopolysaccharide assembly protein B	Tetratricopeptide repeat	NU		
QDX16500.1	putative fimbrial chaperone YadV (plasmid)	Pili and flagellar-assembly chaperone, PapD N-terminal domain	NU		
AVP34824.1	Oligopeptide transport system permease protein OppC	ABC-type dipeptide oligopeptide nickel transport systems permease components	EP		
AVP33889.1	Ketol-acid reductoisomerase (NADP(+)	ketol-acid reductoisomerase activity	EH		
AVP32856.1	hypothetical protein C6W84_00190	Not Detected			
AVP33009.1	hypothetical protein C6W84_01200	Not Detected			
AVP33028.1	hypothetical protein C6W84_01320	Not Detected			
AVP33176.1	hypothetical protein C6W84_02305	Not Detected			
AVP33177.1	hypothetical protein C6W84_02310	Not Detected			
AVP33248.1	hypothetical protein C6W84_02790	Not Detected			
AVP33279.1	hypothetical protein C6W84_03020	Not Detected			
AVP33373.1	hypothetical protein C6W84_03620	Not Detected			
AVP33580.1	hypothetical protein C6W84_05085	Not Detected			
AVP33692.1	hypothetical protein C6W84_05820	Not Detected			

AVP33693.1	hypothetical protein C6W84_05825	Not Detected			
AVP33699.1	hypothetical protein C6W84_05865	Not Detected			
AVP33802.1	hypothetical protein C6W84_06525	Not Detected			
AVP33804.1	hypothetical protein C6W84_06535	Not Detected			
AVP34186.1	hypothetical protein C6W84_09235	Not Detected			
AVP34268.1	hypothetical protein C6W84_09800	Not Detected			
AVP34313.1	hypothetical protein C6W84_10080	Not Detected			
AVP34423.1	hypothetical protein C6W84_10805	Not Detected			
AVP34425.1	hypothetical protein C6W84_10815	Not Detected			
AVP34632.1	hypothetical protein C6W84_12180	Not Detected			
AVP34767.1	hypothetical protein C6W84_13010	Not Detected			
AVP35073.1	hypothetical protein C6W84_15080	Not Detected			
AVP35178.1	hypothetical protein C6W84_15815	Not Detected			
AVP35249.1	hypothetical protein C6W84_16295	Not Detected			
AVP35266.1	hypothetical protein C6W84_16390	Not Detected			
AVP35269.1	hypothetical protein C6W84_16410	Not Detected			
AVP35311.1	hypothetical protein C6W84_16710	Not Detected			
AVP35340.1	hypothetical protein C6W84_03585	Not Detected			
AXU44227.1	hypothetical protein C6W84_4540	Not Detected			
AXU44229.1	hypothetical protein C6W84_4550	Not Detected			
QDX16346.1	hypothetical protein C6W84_16836 (plasmid)	Not Detected			
QDX16454.1	hypothetical protein C6W84_16874 (plasmid)	Not Detected			
AXU43671.1	hypothetical protein C6W84_1760	Not Detected			
AXU43832.1	hypothetical protein C6W84_2565	Not Detected			
AXU44012.1	hypothetical protein C6W84_3465	Not Detected			
QDX16345.1	hypothetical protein C6W84_16835 (plasmid)	Not Detected			
QDX16527.1	hypothetical protein C6W84_16947 (plasmid)	Not Detected			
QDX16528.1	hypothetical protein C6W84_16948 (plasmid)	Not Detected			
AXU43800.1	hypothetical protein C6W84_2405	Not Detected			
AVP35177.1	hypothetical protein C6W84_15810	Not Detected			
AVP34770.1	hypothetical protein C6W84_13030	Not Detected			
AVP34616.1	hypothetical protein C6W84_12080	Not Detected			
AVP34515.1	hypothetical protein C6W84_11490	Not Detected			
AVP33197.1	hypothetical protein C6W84_02440	Not Detected			
AVP33179.1	hypothetical protein C6W84_02335	Not Detected			
AVP33107.1	hypothetical protein C6W84_01910	Not Detected			
AVP32961.1	hypothetical protein C6W84_00880	Not Detected			
AVP32904.1	hypothetical protein C6W84_00515	Not Detected			

Table S2: List of TonB Dependent Transporters (TonRs) detected in OMVs of *A. baumannii* DS002.

Accession no. (gi no)	Protein name	Gene name	Mol. weight (kDa)	PI	sequence coverage (%)	#Peptides
1472139223	putative TonB-dependent receptor BfrD	*bfrD_1*	67.1	6.96	58	36
1472139224	putative TonB-dependent receptor BfrD	*bfrD_2*	19.7	8.94	52	11
1367986576	putative TonB-dependent receptor BfrD	*bfrD_3*	77.9	6.15	48	36
1367985001	Vitamin B12 transporter BtuB	*btuB_1*	67.9	5.27	68	43
1367985595	Vitamin B12 transporter BtuB	*btuB_2*	77.4	6.19	37	25
1472139738	Vitamin B12 transporter BtuB	*btuB_3*	45	5.2	9	3
1472138795	hypothetical protein C6W84_0320	*C6W84_0320*	16.7	5.07	9	1
1472138814	putative TonB-dependent receptor	*C6W84_0415*	16.7	5.07	8	3
1367986745	hypothetical protein C6W84_13005	*C6W84_13005*	14.2	5.24	67.5	6
1367987134	Colicin I receptor	*cirA_2*	100.8	7.66	5	3
1472139004	Ferrichrome receptor FcuA	*fcuA_1*	26	6.55	15	4
1472139005	Ferrichrome receptor FcuA	*fcuA_2*	41.4	6.8	28	7
1714623787	Fe (3+) dicitrate transport protein FecA (plasmid)	*fecA*	77.2	5.47	57	29
1367985635	Fe (2+) transporter FeoB	*feoB*	67.5	7.05	2	1
1472139108	Ferrichrome-iron receptor	*fhuA_3*	80.1	6.02	11	6
1367986193	Ferrichrome-iron receptor	*fhuA_4*	78.2	5.81	37	21
1714623831	Ferrichrome-iron receptor (plasmid)	*fhuA_5*	76.6	5.47	43	32
1472139573	FhuE receptor	*fhuE_1*	52.3	4.91	4	1
1472139711	FhuE receptor	*fhuE_2*	79.2	5.06	25	14
1472139574	Ferripyoverdine receptor	*fpvA*	11.7	8.88	10	1
1472138796	Ferric aerobactin receptor	*iutA_1*	63.3	5.82	14	8
1367987404	Ferric aerobactin receptor	*iutA_3*	80.7	5.35	53	37
1367986134	Ferric enterobactin receptor	*pfeA*	82.8	5.78	15	11
1472139893	Ferri-bacillibactin esterase BesA	*besA*	35.6	8.9	5	1

**Strains**	**Relevant characteristics**	**Reference or source**
Acinetobacter baumannii DS002	Wild type strain. Cmr, Smr	Longkumer et al. 2013
E. coli. DH5 α	λsupE44, ΔlacU169 (Δ80 lacZΔM15) hsdR17 recA1 endA1 gyrA96 thi1 relA1	Hanahan 1983
E. coli. NiCo21(DE3)	BL21(DE3) glmS6Ala slyD-CBD can-CBD arnA-CBD	Robichon et al. 2011
**Plasmids**		
pET28a	Km^r, Expression Vector	Novagen.
pGD1	Km^r, Expression plasmid, Codes for $OmpA^{N6xHis}$	This study
pTRW1	Amp^r, Expression Plasmid, Codes for $RepA^{N6xHis}$	Longkumer et al. 2013
**Primers**		
GD1 FP	GCCTCA*CATATG*AAATTGAGTCGTATTGCACTTG	Primers used to amplify *ompA* gene as *NdeI* and *XhoI* fragment
GD1 RP	TTGACT*CTCGAG*TTGAGCTTGTTGACCAGGTT	

Table S3: List of Strains, plasmids and primers.

Table S4: Gradient used for separating peptides.

Gradient: SolnA-5%ACN+.1%FA   SolnB-95%ACN+0.1%FA

Time [mm:ss]	Duration [mm:ss]	Flow [nl/min]	Mixture [%B]
00:00	00:00	300	0
38:00	38:00	300	25
45:00	07:00	300	40
50:00	05:00	300	90
55:00	05:00	300	90
60:00	05:00	300	3
